# *Schoenoplectus californicus* (C.A. Mey.) Soják: Chemical Profile, Antioxidant Capacity, Psychopharmacological Exploration and Analgesic Activity

**DOI:** 10.3390/md24050160

**Published:** 2026-04-30

**Authors:** Julio Campos-Florián, Gladys Galliani-Huamanchumo, Alessandra Victoria Campos-Bazán, Betsabé Chunga-Flores, Inés Castro-Dionicio, Víctor E. Villarreal-La Torre, Lucia Fátima Flores-Atoche, Lucia Gonzales-Mendez, Gianfranco Ramos-Farfán, José Condor-Goytizolo, Ana María Guevara-Vásquez, Marilú Roxana Soto-Vásquez, Juan Carlos Rodríguez-Soto, Paul Alan Arkin Alvarado-García, William Sagástegui-Guarniz, Billy Cabanillas-Amado

**Affiliations:** 1 Department of Pharmacology, Faculty of Pharmacy and Biochemistry, Universidad Nacional de Trujillo, Trujillo 13011, Peru; bchungaf@unitru.edu.pe (B.C.-F.); vvillarreal@unitru.edu.pe (V.E.V.-L.T.); aguevara@unitru.edu.pe (A.M.G.-V.); wsagastegui@unitru.edu.pe (W.S.-G.); 2 Faculty of Pharmacy and Biochemistry, Universidad Nacional de Trujillo, Trujillo 13011, Peru; ggalliani@unitru.edu.pe (G.G.-H.); acamposb@unitru.edu.pe (A.V.C.-B.); lfloresa@unitru.edu.pe (L.F.F.-A.); lgonzalesm@unitru.edu.pe (L.G.-M.); gramos@unitru.edu.pe (G.R.-F.); jcondor@unitru.edu.pe (J.C.-G.); 3 Pharmacology of Natural Products Group, Universidad Nacional de Trujillo, Trujillo 13011, Peru; 4 Laboratory for Research in the Pharmacology of Natural Products, Universidad Nacional de Trujillo, Trujillo 13011, Peru; 5 EGENODIA Inserm U1283, CNRS UMR8199, Institute Pasteur de Lille, Lille University Hospital, Université de Lille, 59045 Lille, France; ines.castro@univ-lille.fr; 6 Bioinformatic and Cheminformatics Group, Universidad Nacional de Trujillo, Trujillo 13011, Peru; 7 Natural Products and Bioactive Substances Research Group, Faculty of Pharmacy and Biochemistry, Universidad Nacional de Trujillo, Trujillo 13011, Peru; msoto@unitru.edu.pe; 8 Biological Sciences Faculty, Universidad Nacional de Trujillo, Trujillo 13011, Peru; jrodriguezs@unitru.edu.pe; 9 School of Psychology, Faculty of Health Sciences, Universidad Autónoma del Perú, Lima Sur Campus, Lima 15842, Peru; palvaradog@autonoma.edu.pe; 10 Laboratorios de Investigación y Desarrollo, Facultad de Ciencias e Ingeniería, Universidad Peruana Cayetano Heredia, Lima 15102, Peru; billy.cabanillas.a@upch.pe

**Keywords:** analgesic, anxiolytic, antioxidant activity, central nervous system, Schoenoplectus californicus, LC–MS/MS, phytochemicals, in silico prediction

## Abstract

*Schoenoplectus californicus*, a macrophyte from Peruvian marine–coastal wetlands, is traditionally used for medicinal purposes, yet its pharmacological potential remains insufficiently explored. This study evaluated the chemical profile, antioxidant capacity, psychopharmacological effects, and analgesic activity of a hydroethanolic extract from its rhizomes. Phytochemical screening and LC–MS/MS analyses were performed to characterize secondary metabolites. Antioxidant activity was assessed using DPPH and ABTS assays, while in vivo anxiolytic, sedative, and analgesic effects were evaluated in Balb/c mice through open field, elevated plus maze, rotarod, analgesimeter, tail-flick, and hot plate tests, with diazepam and tramadol as reference drugs. In silico PASS and BOILED-Egg analyses were used to predict pharmacological mechanisms and central nervous system permeability. The extract contained flavonoids, phenolic compounds, and stilbenes and exhibited notable antioxidant activity (IC_50_: 0.7319 mg/mL for DPPH and 0.6207 mg/mL for ABTS). Anxiolytic effects were observed at 50 mg/kg, sedative effects at 200 mg/kg, and significant analgesic activity at 50 mg/kg. Several compounds were predicted to cross the blood–brain barrier, with inhibition of GABA aminotransferase suggested as a potential mechanism. Acute toxicity was detected (LD_50_ > 2000 mg/kg). These findings support *S. californicus* as a promising source of neuroactive and analgesic compounds, although further mechanistic and dose-optimization studies are required.

## 1. Introduction

Peru is internationally recognized as one of the world’s 17 megadiverse countries, a status defined by its wide array of ecosystems shaped by diverse climatic and edaphic conditions, encompassing Amazonian rainforests, the high Andes, coastal deserts, and wetlands [1]. In recent years, marine–coastal ecosystems, particularly wetlands, have attracted increasing scientific attention due to their ecological relevance for biodiversity conservation and their potential as sources of bioactive compounds with biotechnological and medicinal applications [2]. Peru hosts approximately 92 wetlands, four of which are located in the La Libertad region. These ecosystems are dominated by the aquatic plant *Schoenoplectus californicus* (C.A. Mey.) Soják, commonly known as “totora” or “junco”, a member of the Cyperaceae family native to the Americas [3,4,5].

This perennial emergent macrophyte and facultative halophyte can reach heights of up to 4 m and thrives in lakes, marshes, flooded areas, and marine–coastal environments from North America to Patagonia and several Pacific islands. Owing to its high primary productivity and rapid growth rate, *S. californicus* is capable of lateral expansion of up to 3 m per season under optimal conditions [6,7]. Beyond its ecological role, the species has held considerable cultural and socioeconomic importance in Peru; pre-Columbian civilizations such as the Moche and Chimú employed totora in the construction of traditional reed boats (“caballitos de totora”), dwellings, floating islands, handicrafts, and everyday tools [3,8].

From a phytochemical perspective, several studies on related genera such as Cyperus and Scirpus have reported the presence of flavonoids (e.g., quercetin, myricetin, luteolin), phenolic acids (e.g., p-coumaric and caffeic acids), stilbenes such as trans-resveratrol, tannins, and other polyphenols [9,10]. Additional compounds, including phenolic glycosides and aromatic amines such as tyramine and N-feruloyltyrosine, have also been described [11]. In *S. californicus*, available evidence indicates the occurrence of reducing sugars, lipids, amino acids such as L-tyrosine, and nucleosides including adenosine. Taken together, these findings suggest that this macrophyte contains multiple classes of secondary metabolites—phenolics, flavonoids, and amines—commonly associated with antioxidant and other biological activities [9,11].

Despite this growing body of phytochemical information, the pharmacological potential of *S. californicus*, particularly with respect to central nervous system (CNS) activity and analgesic effects, remains insufficiently explored. Traditional medicine reports the use of totora in the treatment of ailments such as athlete’s foot, fever, and colds, suggesting the presence of bioactive secondary metabolites [12,13]. This assumption is supported by studies on related *Schoenoplectus* species, in which phenolics, flavonoids, tannins, and terpenoids—compounds frequently linked to antioxidant, analgesic, and sedative properties—have been identified in rhizome extracts [12,14]. However, a point of debate in the field concerns the real in vivo relevance of these metabolites: while some authors argue that the limited bioavailability of polyphenols restricts their impact on CNS function, others report measurable neuropharmacological and analgesic effects even at low systemic concentrations, highlighting the need for further experimental clarification.

Previously, our group demonstrated that a hydroethanolic extract of *S. californicus* exhibits radical scavenging activity and modulates locomotor behavior in murine models [12]. Building on these findings and its ethnomedicinal background, the present study aimed to evaluate the central nervous system activity and analgesic potential of an hydroethanolic extract from *S. californicus* rhizomes using in vivo experimental models complemented by predictive in silico analyses and chemical profile of the extract by LC–MS.

## 2. Results

### 2.1. Preliminary Phytochemical Screening and Metabolic Profiling

To establish a chemical baseline and guide the subsequent molecular identification, a qualitative phytochemical screening of the hydroethanolic extract from *Schoenoplectus californicus* rhizomes was performed (Table 1). This preliminary approach in natural product research, confirmed the presence of key bioactive families, specifically flavonoids, phenolic compounds (high intensity), amino acids, amines, tannins (moderate intensity), reducing sugars, and fats. Conversely, negative results were obtained for alkaloids, triterpenes, steroids, anthocyanins, lactones, quinones, cardiotonic glycosides, saponins, resins, catechins, and polysaccharides.

### 2.2. LC-MS/MS and Molecular Networking Analysis

To comprehensively explore the chemical diversity of *S. californicus* rhizomes, a molecular network was constructed from the acquired LC-MS/MS data. The FBMN analysis of the LC-MS data acquired in positive ion mode yielded 451 nodes, of which 168 were connected in clusters of at least two nodes. As a result, different molecular families were identified, including hydroxycinnamic acid derivatives, stilbenes, proanthocyanidins, N-acetyl ethanolamines, chalcones, and catechins (Figures S1 and S2).

Fifteen compounds were identified using the spectral library search module from MZmine, corresponding to MSI Level 2 annotations, with some Level 3 assignments reflecting unresolved isomeric ambiguity (e.g., sucrose and catechin) or limited MS/MS fragmentation (adenosine). These annotated compounds are summarized in Table 2, which includes diverse metabolites such as catechin (flavan-3-ol), dibenzylamine (phenylmethylamine), xanthoxylin (alkyl-phenylketone), and 4-methylumbelliferyl glucoside (coumarin), among others. Complementarily, additional detected features and related compounds are presented in Table 1 and Table S1, providing a broader overview of the metabolite profile identified in this species.

Given the relevance of stilbenes in *S. californicus*, a more detailed annotation of this chemical family was performed by comparing experimental MS/MS spectra in negative ion mode with reference spectra reported in the literature. The results of this targeted analysis are presented in Table 3, where major stilbenes such as piceatannol, scirpusin A, and scirpusin B were identified based on their retention times, accurate mass measurements, low ppm error, and characteristic fragmentation patterns, corresponding to MSI Level 3 annotation. These compounds have been previously reported for this species, supporting the reliability of their identification.

Furthermore, eighteen additional compounds were annotated using SIRIUS 5.8.5 software (Table S2). These compounds are distinct from those reported in Table 2 and Table 3; however, several of their chemical families (e.g., gneyulin B, N-feruloyl-3-methoxytyramine) matched those of the metabolites identified through spectral matching within the same molecular network cluster. Accordingly, these annotations are considered MSI Level 3, supporting the usefulness of SIRIUS as a complementary tool for the tentative annotation of features, particularly at the chemical family level.

Diverse other compounds such as catechin (flavan-3-ol), dibenzylamine (phenylme-thylamine), xanthoxylin (alkyl-phenylketone), 4-methylumbelliferyl glucoside (coumarin) and other identified features are presented in Table 2 and Table S1.

### 2.3. Antioxidant Capacity

The antioxidant capacity of the hydroethanolic extract from *Schoenoplectus californicus* rhizomes was evaluated using DPPH and ABTS radical scavenging assays. Results are summarized in Table 4. The IC_50_ value for the DPPH assay was 0.7319 ± 0.0098 mg/mL, while the ABTS assay yielded an IC_50_ of 0.6207 ± 0.0062 mg/mL. These IC_50_ values indicate moderate to strong free radical scavenging potential, comparable to natural antioxidants in related plant species. Trolox was used as a commercial reference standard, exhibiting IC_50_ values of 0.1793 mg/mL ± 0.0038 mg/mL and 0.1796 mg/mL ± 0.0059 mg/mL for DPPH and ABTS, respectively. Although the crude extract shows higher IC_50_ values than the pure standard, its radical scavenging potential is considered significant given the complex nature of the hydroethanolic matrix.

### 2.4. Central Nervous System Activity

#### 2.4.1. Open Field

**Figure 1:**
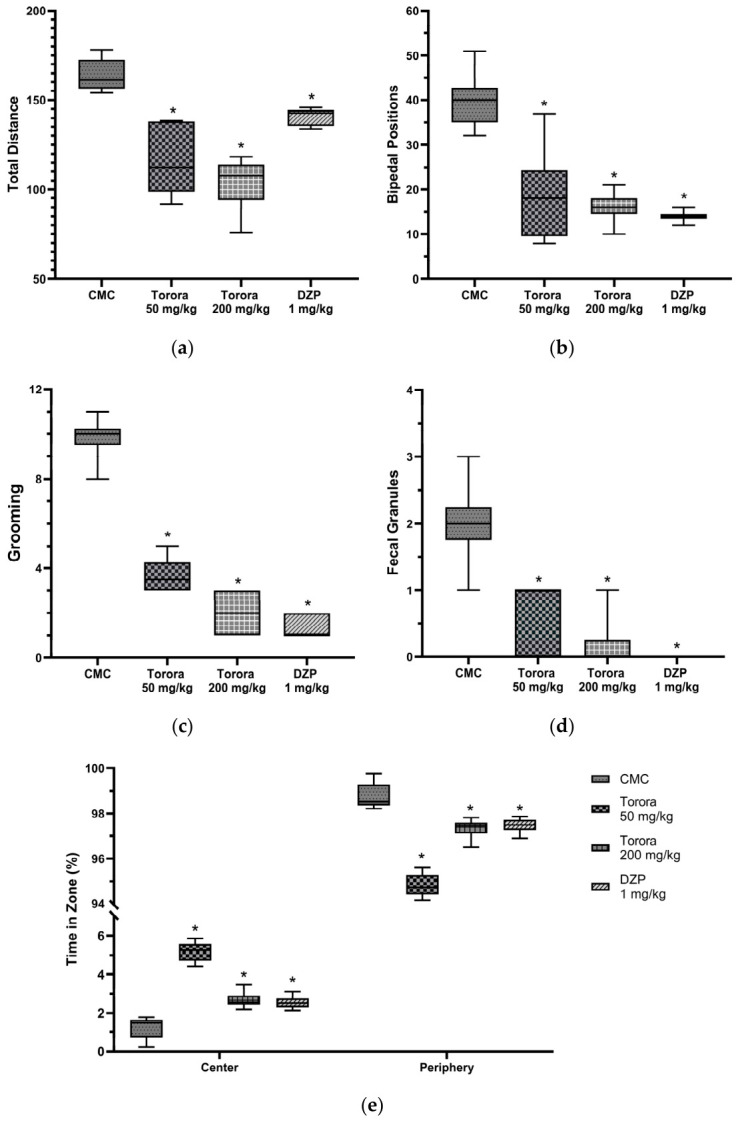
Open Field test. Average values of the number of total distance (**a**), bipedal positions (**b**), grooming (**c**), fecal granules (**d**) and time in zone (%) (**e**) in the open field test according to the experimental group. CMC group received carboxymethylcellulose 0.5%, DZP group received diazepam 1 mg/kg. Data are expressed as mean ± SEM (*n* = 7). * *p* < 0.05 vs. CMC (one-way ANOVA followed by Tukey’s post hoc test).

The open field test was used to evaluate behavioral parameters related to locomotion, exploration, and anxiety-like responses, as shown in Figure 1 and Figure S4. The hydroethanolic extract of *Schoenoplectus californicus* rhizomes (Totora, 50 mg/kg and 200 mg/kg) as well as diazepam (DZP, 1 mg/kg) significantly modified (*p* < 0.05) these parameters compared to the vehicle control (CMC, 0.5% carboxymethylcellulose). In Figure 1, regarding total distance traveled (a), a statistically significant reduction was observed between CMC and DZP, with both Totora doses producing an even greater decrease than DZP and markedly lower values compared to the CMC group. Concerning bipedal positions (b), both Totora and DZP significantly reduced this behavior, with Totora at 200 mg/kg resembling DZP most closely. Grooming activity (c) was also significantly diminished by both treatments, with the higher Totora dose showing a profile similar to DZP. In terms of excretions (d), a large number of fecal pellets were observed in the CMC group, whereas this parameter was absent in the DZP group and almost negligible in both Totora doses, closely approximating the anxiolytic control. Finally, analysis of time spent in different zones of the arena (Figure 1e and Figure S4) mice in the CMC group (Figure S4a) showed minimal permanence in the central zone and a clear preference for the periphery, together with one of the highest values of total distance traveled, a behavioral pattern indicative of increased anxiety. In contrast, the DZP group and the Totora 200 mg/kg group exhibited slightly higher values of time spent in the central zone (Figures S2d and S4c). Notably, the Totora 50 mg/kg group showed the highest permanence in the central zone of the open field (Figure 1e and Figure S4b). Overall, the hydroethanolic extract of *Schoenoplectus californicus* at doses of 50 mg/kg and 200 mg/kg produced measurable changes in exploratory behavior and locomotor activity.

#### 2.4.2. Elevated Plus Maze

**Figure 2:**
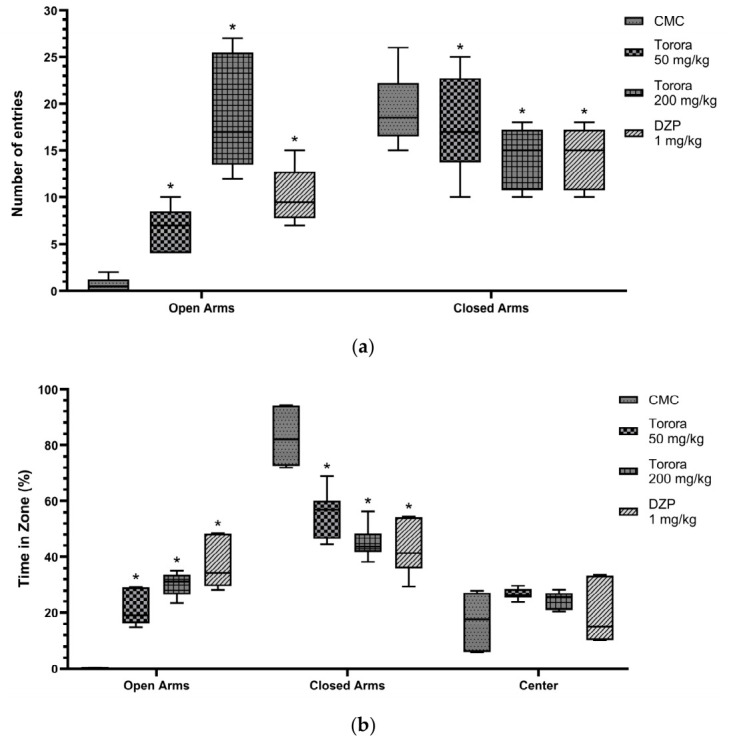
Elevated Plus Maze test. Values expressed in number of entries in open and closed arms (**a**); and in percentages of the time spent in open arms, closed arms and central square (**b**) of the elevated plus maze according to experimental group. CMC group received carboxymethylcellulose 0.5%, DZP group received diazepam 1 mg/kg. Data are expressed as mean ± SEM (*n* = 7). * *p* < 0.05 vs. CMC (two-way ANOVA followed by Tukey’s post hoc test).

The elevated plus maze (EPM) assessed anxiety-like behaviors through entries into and time spent in open versus closed arms. Compared to the vehicle control (CMC), the hydroethanolic extract of *Schoenoplectus californicus* rhizomes (Totora, 50 mg/kg and 200 mg/kg) and diazepam (DZP, 1 mg/kg) significantly modulated (*p* < 0.05) these parameters. In Figure 2, regarding the number of entries (a), statistical significance was observed, with the CMC group showing almost no entries into the open arms, whereas Totora at 200 mg/kg produced the highest number of entries, surpassing even DZP, which in turn exceeded Totora 50 mg/kg; this behavioral pattern is visually corroborated in Figure S5, where the trajectories of rodents treated with CMC 0.5% reveal an evident avoidance of the open arms (Figure S5a), in contrast to the exploratory paths of animals receiving Totora 200 mg/kg or DZP (Figure 2a), which demonstrate increased entries into these zones (Figure S5c,d). In closed arm entries, statistical differences were also evident, although no major variation was observed among groups. Concerning the time spent in the different zones (Figure 2b), DZP led the permanence in the open arms, followed by Totora 200 mg/kg and then Totora 50 mg/kg, while the CMC group spent the longest duration in closed arms and markedly less in the open ones. Time spent in the central square showed no substantial differences among groups.

#### 2.4.3. Rotarod Test

**Figure 3:**
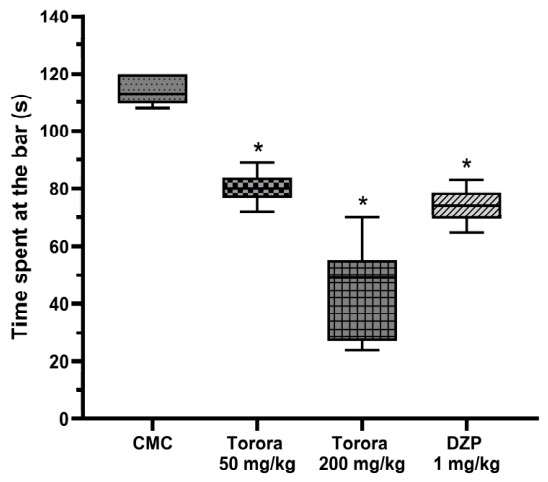
Rotarod test. Values expressed in seconds of the time spent on the rotarod bar according to the experimental group. CMC group received carboxymethylcellulose 0.5%, DZP group received diazepam 1 mg/kg. Data are expressed as mean ± SEM (*n* = 7). * *p* < 0.05 vs. CMC (one-way ANOVA followed by Tukey’s post hoc test).

The rotarod test evaluated motor coordination and balance as an indicator of potential sedative or muscle relaxant effects. In Figure 3, compared to the vehicle control (CMC), the hydroethanolic extract of *Schoenoplectus californicus* rhizomes (Totora 50 mg/kg and 200 mg/kg) and diazepam (DZP, 1 mg/kg) significantly (*p* < 0.05) reduced time spent on the rotating bar.

These reductions suggest mild impairment in motor coordination with the extract, though more pronounced than with Totora 200 mg/kg, indicating a potential sedative effect without severe neurotoxicity.

### 2.5. Analgesic

**Figure 4:**
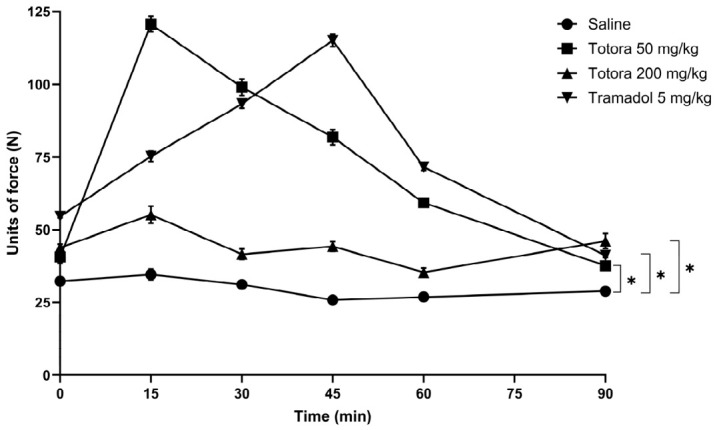
Analgesimeter Test. Average values expressed in force units (N) in the analgesimeter test according to the experimental group. Data are presented as the mean ± SEM (*n* = 7). Statistical significance was assessed by two-way ANOVA followed by Tukey’s HSD post hoc test: * *p* < 0.05 vs. saline group.

**Figure 5:**
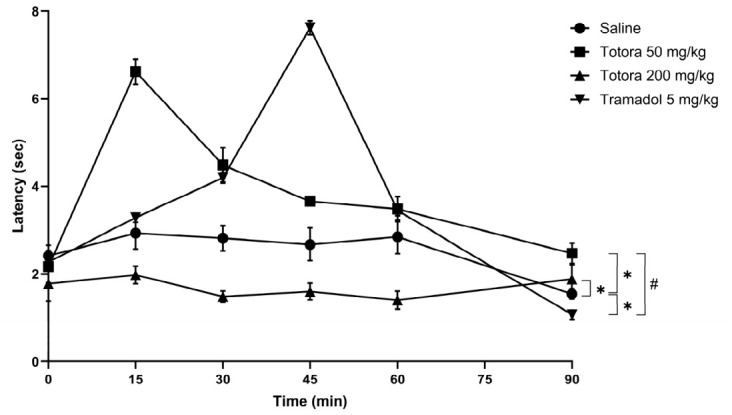
Tail Withdrawal Test. Average values of reaction time expressed in seconds (sec) in the tail removal test according to the experimental group. Data are presented as the mean ± SEM (*n* = 7). Statistical significance was assessed by two-way ANOVA followed by Tukey’s HSD post hoc test: * *p* < 0.05 vs. saline group; # *p* > 0.05 vs. Tramadol group.

**Figure 6:**
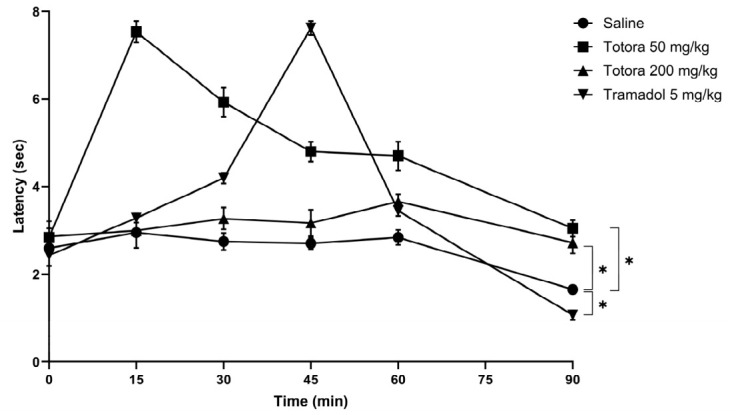
Hot Plate Test. Average reaction time values expressed in seconds (sec) in the Hot Plate test according to the experimental group. Data are presented as the mean ± SEM (*n* = 7). Statistical significance was assessed by two-way ANOVA followed by Tukey’s HSD post hoc test: * *p* < 0.05 vs. saline group.

The antinociceptive potential of the hydroethanolic extract from *Schoenoplectus californicus* rhizomes was evaluated in male Balb/c mice using mechanical (Analgesimeter) and thermal (Tail-Flick and Hot Plate) nociception assays (Figure 4, Figure 5 and Figure 6). In all tests, the saline group consistently showed the lowest response.

Administration of Totora at 50 mg/kg produced a rapid and pronounced antinociceptive effect across all models. This dose reached its maximal response at 15 min post-administration, with peak values of approximately 121 N in the analgesimeter test (Figure 4), 6.6 s in the tail withdrawal assay (Figure 5) and 7.5 s in the hot plate test (Figure 6). In contrast, tramadol (5 mg/kg) reached its maximal effect later, at 45 min, with peak values of approximately 115 N, 7.6 s, and 7.6 s in the analgesimeter, tail withdrawal and hot plate tests, respectively. While peak magnitudes were comparable in the thermal assays, Totora 50 mg/kg showed a more rapid onset of action.

Importantly, the antinociceptive effect of Totora 50 mg/kg was more persistent over time than that of tramadol in the thermal nociception models, maintaining higher latency values toward the end of the observation period, particularly in the tail withdrawal test (Figure 5). In contrast, the higher dose of Totora (200 mg/kg) failed to elicit a meaningful antinociceptive response, with values remaining close to those of the saline group across all assays.

### 2.6. In Silico Pharmacological Activity Prediction

In silico screening using the PASS system (Way2Drug server) predicted several pharmacological activities for the compounds identified in *Schoenoplectus californicus*. Only predictions in which the probability of activity (Pa) exceeded the probability of inactivity (Pi) were considered.

**Figure 7:**
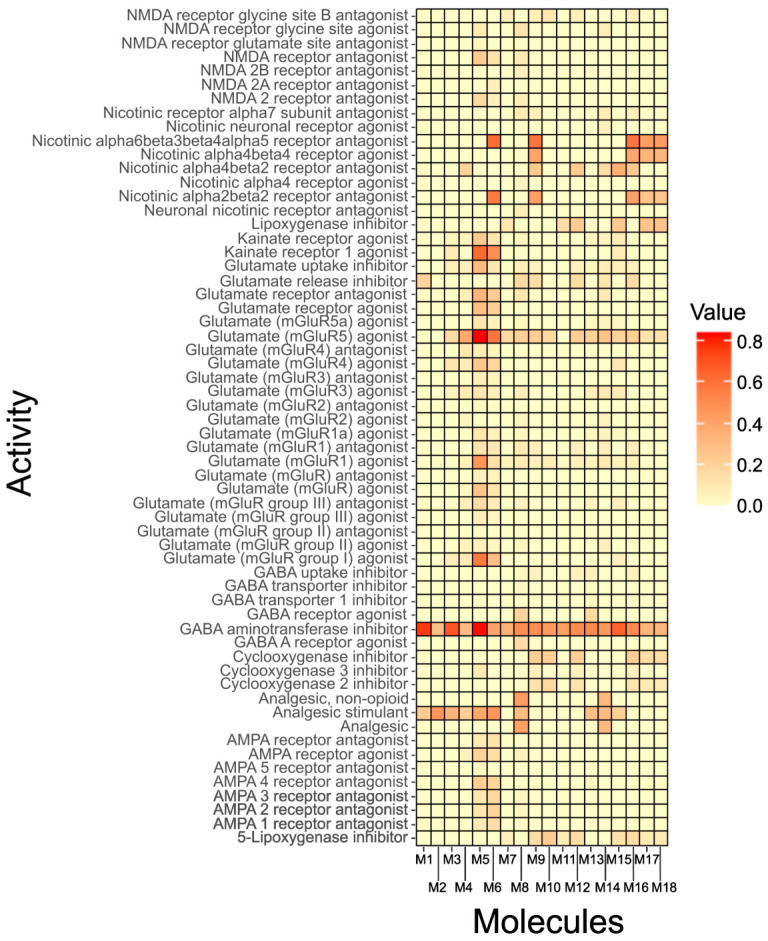
Prediction of activities of 18 molecules detected in *Schoenoplectus californicus*. Value in Prediction activity (Pa). M1: sucrose, M2: adenosine, M3: N-fructosylisoleucine, M4: N,N,N-trimethyl-L-alanine-L-proline betaine, M5: L-tyrosine, M6: L-Tryptophan, M7: D-(+)-catechin, M8: dibenzylamine, M9: xan-thoxylin, M10: kaempferol, M11: N-feruloyltyramine, M12: flavokawain C, M13: lauryldiethano-lamine, M14: 1,3-dicyclohexylurea, M15: 9-Oxo-10E,12Z-octadecadienoic acid, M16: piceatannol, M17: scirpusin B, M18: scirpusin A.

Heatmap visualization of Pa values for the 18 LC-MS/MS–identified metabolites (Figure 7) showed that inhibition of GABA aminotransferase emerged as the most consistently predicted mechanism across the dataset. Although all compounds displayed some degree of predicted activity at this target, L-tyrosine, N-fructosylisoleucine, and Sucrose exhibited the highest Pa values. Additionally, L-tyrosine showed notable probabilities for actions on glutamatergic signaling, including predicted activity as an mGluR5 agonist and Kainate receptor 1 agonist, suggesting a broader neuromodulatory potential.

### 2.7. Prediction of Physicochemical Properties

**Figure 8:**
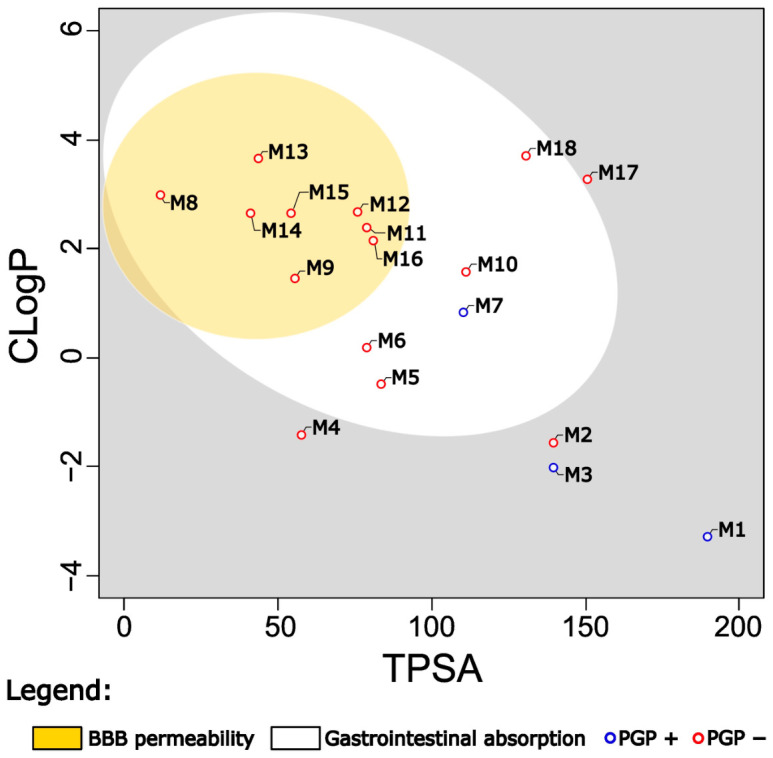
Prediction of Gastrointestinal absorption and Brain Penetration of 18 molecules of *Schoenoplectus californicus*, and Prediction of be P Glicoprotein substrate. M1: sucrose, M2: adenosine, M3: N-fructosylisoleucine, M4: N,N,N-trimethyl-L-alanine-L-proline betaine, M5: L-tyrosine, M6: L-Tryptophan, M7: D-(+)-catechin, M8: dibenzylamine, M9: xan-thoxylin, M10: kaempferol, M11: N-feruloyltyramine, M12: flavokawain C, M13: lauryldiethano-lamine, M14: 1,3-dicyclohexylurea, M15: 9-Oxo-10E,12Z-octadecadienoic acid, M16: piceatannol, M17: scirpusin B, M18: scirpusin A.

Physicochemical properties, including topological polar surface area (TPSA) and consensus log P (CLogP), were predicted for the 18 identified compounds from *Schoenoplectus californicus* using the SwissADME server. These parameters were utilized to generate a Brain Or IntestinaL EstimateD permeation (BOILED-Egg) plot (Figure 8), which models gastrointestinal absorption and blood–brain barrier (BBB) permeability. The analysis revealed that six compounds are predicted to permeate the BBB (yellow region), indicating potential central nervous system activity. Additionally, three compounds exhibited low predicted gastrointestinal absorption (gray region). P-glycoprotein (PGP) substrate status was also evaluated, with catechin and N-fructosylisoleucine identified as the only PGP substrates among the compounds.

### 2.8. Acute Toxicity

The acute oral toxicity of the hydroethanolic extract of *Schoenoplectus californicus* rhizomes was evaluated in Balb/c mice (*n* = 5 per sex), following the recommendations of OECD Guideline 420. Since no toxicity was observed at a dose of 300 mg/kg, the maximum dose of 2000 mg/kg body weight was used. One mouse from each group died on the first day of the trials. Piloerection and tachypnea were observed between the first and second day; thereafter, no clinical signs of toxicity or serious pathological changes were observed during the 14-day monitoring period. Body weight measurements on days 7 and 14 after administration showed only slight changes without significant deviations from the control group (Supplementary File S1—Table S3; full report in original Spanish available in Supplementary File S2). On macroscopic examination, the organs showed no abnormalities, and organ weights showed no significant variations, indicating no organ-specific toxicity (Supplementary File S2). These findings classify the extract as low toxicity (Category 5, LD50 > 2000 mg/kg) under the Globally Harmonized System of Classification and Labelling of Chemicals.

## 3. Discussion

The escalating pursuit of novel pharmacological agents has intensified research into natural sources. Among these, *Schoenoplectus californicus* (C.A. Mey.) Soják stands out as a prominent facultative halophyte and macrophyte, a Cyperaceae species traditionally utilized in Peruvian marine–coastal wetlands [4,8,15]. Given its ecological adaptation to saline-influenced environments, this study conducted a pharmacological screening, including chemical composition analysis, antioxidant capacity, central nervous system (CNS) effects, analgesic activity, and in silico predictions. The research was informed by its longstanding ethnopharmacological uses in coastal communities, particularly for the treatment of conditions associated with malaise and anxiety.

Phytochemical screening (Table 1) revealed the presence of flavonoids, phenolic compounds (high intensity), amino acids/amines, tannins (moderate), reducing sugars, and fats, consistent with prior analyses of *S. californicus* that identified flavonoids and phenols [12]. Related Cyperaceae species, such as *Schoenoplectus triqueter* L. Palla and *Scirpus articulatus*, exhibit similar profiles, including flavonoids, saponins and phenols [12,16,17]. This congruence likely stems from secondary metabolites’ roles as defense mechanisms against evolutionary and ecological stressors characteristic of the marine-coastal wetlands inhabited by *S. californicus*, where plants are subjected to high salinity, intense UV radiation, and variable hydric and oxygen conditions [18,19,20]. These environmental pressures promote the activation of the phenylpropanoid pathway, leading to the accumulation of phenolic acids, flavonoids, and stilbenes that function as antioxidants and osmoprotectants, mitigating oxidative stress and stabilizing cellular membranes [21,22,23]. In halophytic and emergent macrophytes such as *S. californicus*, increased levels of kaempferol, catechins, and hydroxycinnamic acid derivatives have been correlated with their adaptive responses to salinity and radiation [24,25]. This chemical adaptation not only enhances their ecological resilience but also explains the potent antioxidant capacity observed in the rhizome extracts, as evidenced by their low IC_50_ values in the DPPH and ABTS assays (Table 3).

The molecular networking analysis of *S. californicus* rhizomes revealed diverse mo-lecular families, and the use of SIRIUS software enabled the annotation of 18 additional features, demonstrating its capability for tentative identification of additional compounds. While both SIRIUS and spectral library matching provided significant insights, the identification of scirpusin A, scirpusin B, and piceatan-nol; compounds previously documented in this species, was achieved through detailed analysis of their MS/MS spectra [26,27]. This approach underscores the value of combining automated tools with chemotaxonomic data for comprehensive chemical profiling.

Antioxidant assays (Table 4) yielded values indicating strong free-radical scavenging. This activity aligns with the LC–MS/MS identification of flavonoids such as kaempferol and catechin, and the presence of stilbenes in the molecular network of the extract [17,28]. Compared with other species of the same genus, such as *S. triqueter* L. Palla, whose ethyl acetate extract exhibited antioxidant activity in the DPPH radical scavenging assay, *S. californicus* shows greater potency, likely reflecting higher levels and/or diversity of phenolics and flavonoids [12,13]. Collectively, these results underscore species-specific phytochemical differences within Cyperaceae that translate into distinct antioxidant capacities.

CNS evaluations demonstrated that the hydroethanolic extract of *Schoenoplectus californicus* rhizomes exerts dose-dependent anxiolytic and sedative-like effects in male Balb/c mice, with results converging across the open field test, elevated plus maze, and rota rod performance [29,30]. In the open field test, mice treated with the vehicle (CMC) displayed a behavioral pattern characterized by high locomotor activity, minimal permanence in the central zone, and predominant exploration of the periphery, which is widely recognized as an indicator of increased anxiety-like behavior in rodents [31,32]. Avoidance of the central area is one of the most consistent indicators of increased anxiety in rodent models, reflecting their evolutionary aversion to open spaces where they are more exposed to predators [33,34]. In contrast, the extract at 50 mg/kg significantly increased the time spent in the central zone, indicating a clear anxiolytic-like effect without excessive locomotor suppression. At 200 mg/kg, the behavioral profile was similar to diazepam, with reduced locomotion and moderately increased central permanence, consistent with a combined sedative–anxiolytic effect [35,36]. Altogether, these findings confirm the anxiolytic profile of diazepam and demonstrate that Totora, particularly at 200 mg/kg, exhibited comparable or even greater effects in specific parameters, supporting its potential anxiolytic-like activity. These findings were corroborated by the elevated plus maze, where the 200 mg/kg dose increased open-arm exploration, whereas the lower dose showed weaker anxiolytic indices [30,37,38]. The rotarod test further supported a dose-dependent central depressant effect, as both extract doses reduced the time on the rotating bar compared to CMC, although motor impairment was milder than that induced by diazepam [39,40]. Notably, the 200 mg/kg dose produced a greater reduction in motor coordination, while the effect at 50 mg/kg was comparatively milder. Overall, these results indicate a depended dose–response, with anxiolytic effects at lower doses and predominant sedation at higher doses [41,42].

These behavioral outcomes can be attributed to the phytochemical composition of the extract, particularly flavonoids such as kaempferol, catechin, and flavokawain C, which were identified via LC-MS/MS, the flavonoids are known to modulate GABA_A_ receptors, enhancing inhibitory neurotransmission and thereby reducing CNS excitability in a manner analogous to benzodiazepines [43,44,45,46]. This neuropharmacological property provides a plausible explanation for the anxiolytic and sedative-like effects observed in the present study. The depended dose–response pattern is consistent with previous reports on flavonoid-rich plant extracts, such as *Cyperus rotundus* and *Capparis sicula*, where lower doses predominantly elicit anxiolytic effects, while higher doses shift toward sedation, likely due to cumulative CNS depressant activity [16,47,48,49]. In addition to flavonoids, the LC-MS/MS analysis revealed the presence of other phenolic metabolite classes, including proanthocyanidins (e.g., procyanidin B1) and hydroxycinnamic acid derivatives (e.g., N-feruloyltyramine). These compounds are widely recognized for their antioxidant and neuroprotective properties and may contribute to the modulation of oxidative stress and inflammatory processes associated with anxiety-related behaviors [50,51]. The coexistence of these phenolic classes supports a synergistic mode of action, in which multiple metabolites collectively influence neurobehavioral outcomes [52].

Overall, the consistency across behavioral assays supports the dual sedative and anxiolytic potential of the extract, with dose playing a critical role in determining the balance between these effects. At moderate doses, the extract shows clear anxiolytic properties comparable to diazepam, while higher doses predominantly produce sedation, accompanied by mild motor impairment but not severe ataxia [53]. These findings highlight the therapeutic promise of *Schoenoplectus californicus* as a natural sedative–anxiolytic agent, warranting further mechanistic studies to clarify its interaction with GABA receptor subtypes and to optimize dosing strategies that maximize anxiolysis while minimizing sedation [41,46,54].

The prediction of activity demonstrates that the molecules found in *Schoenoplectus californicus* may have inhibitory activity on GABA aminotransferase (Figure 7), an enzyme responsible for metabolizing GABA and which is the site of action of drugs that have demonstrated anxiolytic, antiepileptic, and analgesic activity [55,56]. Although L-tyrosine exhibits a higher propensity for inhibiting GABA aminotransferase, all 18 molecules demonstrate potential activity. Notably, this activity is enhanced across the entire set [46,56]. However, only 7 molecules are capable of crossing the blood–brain barrier, so the enzymatic inhibitory activity could be related to only these molecules (Figure 8). Notably, dibenzylamine and 1,3-dicyclohexylurea should be interpreted with caution. These compounds were detected in the sample but not in the blank solution (LC–MS-grade methanol). As they are not known to be endogenous to plant metabolism, their presence cannot be unequivocally attributed to *S. californicus* biosynthesis and may instead reflect environmental exposure or introduction during sample handling and extraction. Therefore, they are considered non-endogenous features and were not further emphasized in the discussion of biological activity. Similarly, lauryldiethanolamine was detected in the samples and is commonly associated with cosmetic and surfactant formulations; therefore, its presence is considered to be of likely exogenous origin and should be interpreted with caution.

Stilbene such as piceatannol may also be involved in anxiolytic activity. Piceatannol can cross the blood–brain barrier and Yoshizawa et al. (2020) have shown that piceatannol at low concentrations (3 mg/kg) exhibits anxiolytic effects in mice, due to its inhibitory ef-fects on glyoxalase 1 (Glo-I) [54,57].

There is no concrete evidence of the anxiolytic properties of plant species belonging to the genus or botanical family of *Schoenoplectus californicus*. However, plant extracts with high antioxidant capacity can have anxiolytic-like effects due to bioactive compounds such as flavonols, catechins, and chalcones [58,59].

In the analgesimeter test, the dose–response pattern observed indicates that the extract produces analgesic effects at low doses, whereas higher doses are associated with a paradoxical increase in pain sensitivity. This biphasic behavior is frequently observed in plant extracts rich in polyphenols and flavonoids [60,61]. Research has shown that certain flavonoids exhibit hormetic or biphasic effects in pain models, where low doses effectively modulate nociceptive pathways, while higher doses may recruit pro-nociceptive signaling or interfere with other neurotransmitter systems, leading to a reduction in the observed analgesic efficacy [62,63,64].

The antinociceptive effects observed may be attributed to the phytochemical profile of *S. californicus*, particularly its flavonoid content, including kaempferol, catechin, and chalcones such as flavokawain C, which were identified by LC-MS/MS [65,66]. Flavonoids are known to modulate nociceptive processing through central mechanisms independent of inflammatory injury, including interactions with GABAergic, opioidergic, and monoaminergic pathways, as well as modulation of ion channels involved in pain transmission [62,67]. In this context, kaempferol has been reported to exert antinociceptive effects in acute pain models through central signaling rather than anti-inflammatory mechanisms, having various mechanisms such as the inhibition of TLR4 receptors and inhibiting the binding of NF-κβ of DNA and myeloid differentiation factor 88, and suppresses the re-lease of TNF-α, IL- 6, IL-1β, IL-18 [68,69,70].

Consistent with a central mode of action, the hydroethanolic extract also exerted a significant effect on the central nervous system, as evidenced by a marked reduction in locomotor and exploratory activity, as well as anxiety-related behaviors in the open field test. The behavioral profile observed, particularly at 200 mg/kg, was comparable to that of diazepam, indicating a depressant effect on the CNS with modulation of neural circuits involved in anxiety and motor activity. These findings provide convergent evidence that the extract acts centrally, and support the interpretation that the antinociceptive effects observed at lower doses result from neuromodulatory mechanisms, while higher doses may engage opposing pathways, leading to reduced analgesic efficacy and the emergence of hyperalgesia [35,71,72]. The detection of flavokawain C, together with in silico predictions of blood–brain barrier permeability for this compound and other annotated constituents (xanthoxylin, N-feruloyltyramine, 1,3-dicyclohexylurea, lauryldiethanolamine, and dibenzylamine), further supports central exposure to multiple bioactive molecules. Notably, xanthoxylin—also identified in the extract—has documented antinociceptive activity in acute pain models and may contribute to the overall effect through central mechanisms, potentially acting synergistically with flavokawain C, thereby enhancing analgesia at low doses while minimizing hyperalgesia [73,74].

Although limited data exist on the analgesic activity of other compounds in the extract, such as N-feruloyltyramine and lauryldiethanolamine, related dibenzylamine derivatives have shown neurogenic, antioxidant, and neuroprotective activities, suggesting potential contributions to the extract’s overall effects [75,76]. Additionally, 1,3-dicyclohexylurea, a synthetic analog, may exhibit moderate analgesic activity, warranting further investigation. The synergistic interplay of these phytoconstituents, particularly flavonoids and chalcones, likely underlies the extract’s ability to modulate central pain pathways, possibly through interactions with GABA_A_ receptors or opioid receptor systems, as seen in other Cyperaceae species [41,77,78].

The favorable safety profile of *S. californicus*, with an LD50 exceeding 2000 mg/kg in acute toxicity studies, supports its potential as a therapeutic agent. The extract’s antioxidant properties, driven by flavonols and chalcones, further enhance its therapeutic promise by mitigating oxidative stress, a known exacerbator of chronic pain and neuroinflammation. These findings underscore the potential of *S. californicus* as a natural antinociceptive agent with a safer profile than tramadol, which is associated with side effects like sedation and dependency. However, the biphasic dose–response necessitates careful dose optimization to maximize analgesia while minimizing sedation, as observed in higher doses. Future studies should focus on elucidating the specific receptor interactions (e.g., opioid or GABAergic) and conducting pharmacokinetic analyses to confirm the bioavailability and CNS penetration of key phytoconstituents.

## 4. Materials and Methods

### 4.1. Plant Material and Identification

Rhizomes of *Schoenoplectus californicus* (C.A. Mey.) Soják were collected in March 2023 from the marine–coastal wetland located on Huanchaco beach, Trujillo, Peru (8°04′59″ S, 79°07′08″ W). Approximately 2 kg of fresh rhizomes were gathered from 8 to 10 different specimens randomly selected across the study area. The collected material consisted of healthy, mature plants of uniform size, approximately 2 m in height. The rhizomes were cleaned of organic debris at the site. A voucher specimen was authenticated at the Herbarium Truxillense (HUT), National University of Trujillo, and deposited under registration number 59,578 (Figure S6).

### 4.2. Preparation of the Hydroethanolic Extract

Rhizomes were washed, dried at 40 °C (Memmert GmbH + Co. KG, Schwabach, Germany), milled, and sieved (0.75 mm mesh). A total mass of 64.129 g of dry pulverized material (obtained from 570.4 g of fresh rhizomes) was used to prepare a 5% (*w*/*v*) hydroethanolic extract using 50% ethanol (approx. 1.28 L). The mixture was stirred for 16–24 h, filtered, and concentrated under reduced pressure using a rotary evaporator (Laborota 4000, Heidolph Instruments GmbH & Co. KG, Schwabach, Germany). The residue was reconstituted in ultrapure water, transferred to Falcon tubes, and lyophilized after 24 h using a freeze dryer (Millrock Technology BT85, Millrock Technology Inc., Kingston, NY, USA) [79]. After lyophilization, 4.7634 g of dry crude extract was obtained, representing an extraction yield of 7.427% (*w*/*w*) relative to the dry starting material.

### 4.3. Preliminary Phytochemical Screening

Secondary metabolites were identified using standard qualitative methods previously described in the literature [80,81]. The screening included tests for alkaloids (Dragendorff, Wagner, Mayer), triterpenes/steroids (Liebermann–Burchard), flavonoids (Shinoda), anthocyanins, lactones (Baljet), phenolics (ferric chloride), amino acids/amines (ninhydrin), quinones (Borntrager), cardiotonic glycosides (Kedde), saponins (foam test), tannins (gelatin), resins, reducing sugars (Fehling), catechins, fats (Sudan), and polysaccharides (mucilage) [6,79].

### 4.4. LC–MS/MS Analysis

A 1 mg/mL solution of the lyophilized extract was prepared in LC–MS grade methanol and filtered through a 0.2 µm PTFE membrane. Analyses were performed on an Ultimate 3000 UHPLC system coupled to a Q-Exactive Plus mass spectrometer equipped with a heated electrospray ionization (HESI) source (Thermo Scientific, Bremen, Germany). Instrument control was achieved using Xcalibur 4.0 software.

Chromatographic separation was carried out on a Luna Omega C18 column (150 × 2.1 mm, 1.6 µm; Phenomenex, Torrance, CA, USA) at 40 °C, with a 2 µL injection volume and a flow rate of 0.25 mL/min. The mobile phases consisted of 0.1% formic acid in water (A) and acetonitrile (B), using the following gradient: 10% B (0–1 min), 10–100% B (1–19 min), 100% B (19–21 min), 100–10% B (21–22 min), and 10% B (22–27 min).

The mass spectrometer operated in positive ion mode with the following parameters: sheath gas 48 a.u., auxiliary gas 2 a.u., spray voltage +3.0 kV, capillary temperature 300 °C, heater temperature 400 °C, and S-lens RF level 70 a.u. Full MS scans (*m*/*z* 180–1800) were acquired at a resolution of 70,000, and MS/MS scans of the six most intense ions were obtained at 17,500 resolution. 100 ms for Full MS scans, and at 1 × 105 and 50 ms for MS/MS scans, respectively. The MS/MS scans were performed on the six most intense ions detected in full-scan MS (Top6) and the stepped normalized collision energy (NCE) was set to 20, 40, and 50 units. Calibration was performed with Pierce LTQ Velos ESI (Thermo Scientific, Waltham, MA, USA) solution. A blank solution (LC-MS grade methanol) was injected prior to conducting three injections on the sample.

### 4.5. Molecular Networking and Metabolite Annotation

Raw LC–MS/MS data were converted to mzXML format using MSConvert (ProteoWizard v3.0.x, Palo Alto, CA, USA) [82] and processed with MZmine 3.9.0 [83] for peak detection, alignment, and deconvolution (Supplementary Table S1). Spectral library matching was performed against the GNPS database using a precursor and fragment tolerance of 0.02 Da and a minimum of six matched peaks. A weighted cosine similarity score ≥ 0.65 was required.

Processed files (mgf and .csv) were analyzed via the GNPS web platform’s feature-based molecular networking module [84]. Parameters included 0.02 Da mass tolerances for precursor and fragment ions, a minimum cosine score of 0.7, and at least six matched peaks. Additional edges were incorporated to enhance network connectivity. Molecular networks were visualized using Cytoscape 3.8.2 [85].

Additional annotations were performed with SIRIUS 5.8.5 [86], setting a maximum *m*/*z* deviation of 5 ppm for molecular formula prediction, refined by the ZODIAC module. In silico structure annotation utilized COCONUT, GNPS, and Natural Products databases via CSI:FingerID. Systematic chemical class annotations were generated using CANOPUS.

### 4.6. In Vitro Antioxidant Activity

#### 4.6.1. DPPH Radical Scavenging Assay

Antioxidant capacity was evaluated using a modified DPPH method [87]. Extracts (0.001–1 mg/mL) were mixed with 300 µL of DPPH solution in triplicate in 96-well plates and incubated for 15 min in darkness. Absorbance was measured at 517 nm using a microplate reader (Agilent BioTek Epoch, Winooski, VT, USA). Percentage inhibition and IC_50_ values were calculated based on a Trolox (Sigma-Aldrich, St. Louis, MO, USA) calibration curve, which also served as a positive control for the antioxidant assays [88,89].

#### 4.6.2. ABTS Radical Cation Decolorization Assay

The ABTS assay was performed following previously described protocols [90,91]. The radical cation was generated by reacting 7 mM ABTS with 2.5 mM potassium persulfate for 16 h. Extracts (0.001–1 mg/mL, 10 µL) were mixed with 300 µL of the ABTS working solution in triplicate on a 96-well microplate, incubated for 5 min, and the absorbance was measured at 734 nm using an Agilent BioTek Epoch Microplate Spectrophotometer (Winooski, VT, USA). Percentage inhibition and IC_50_ values were calculated based on a Trolox (Sigma-Aldrich, St. Louis, MO, USA) calibration curve, which also served as a positive control for the antioxidant assays [91,92].

### 4.7. Experimental Animals

Thirty-eight Balb/c mice (*Mus musculus*, 45–55 g) were obtained from the vivarium of the National Institute of Health, Lima, Peru. Twenty-eight male mice were used for analgesic assays and, after a two-week washout period, the same animals were employed in central nervous system (CNS) behavioral studies. An additional group of ten mice (mixed sexes) was used exclusively for acute toxicity evaluation.

Animals were acclimatized for 15 days under controlled conditions (12 h light/dark cycle, 23–25 °C), with *ad libitum* access to standard diet and water.

### 4.8. In Vivo CNS Behavioral Studies

Twenty-eight male Balb/c *Mus musculus* (45–55 g) were divided into four groups (*n* = 7): vehicle (0.5% carboxymethylcellulose, 5 mL/kg, oral), diazepam (Valium^®^, Roche; 1 mg/kg, oral), and hydroethanolic extract of *S. californicus* rhizomes (50 or 200 mg/kg, oral). The vehicle solution was prepared using carboxymethylcellulose powder dispersed in ultrapure water in the appropriate proportions to obtain a 0.5% suspension. Both diazepam and the hydroethanolic extract were dissolved in this 0.5% CMC vehicle to achieve their respective dosing concentrations. Thus, the vehicle group received only the CMC suspension, whereas the treated groups received diazepam or extract formulated in the same vehicle. All substances were administered by oral gavage 40 min prior to testing.

#### 4.8.1. Open Field Test

Exploratory behavior, locomotion, and anxiety were evaluated in an open-field apparatus consisting of a completely black square chamber (40 × 40 × 40 cm) [93]. Thirty minutes after treatment administration, each mouse was monitored for 5 min under indirect lighting to avoid glare or stress. Sessions were recorded using a Feeltek Elec HD Webcam (720p) positioned 1.50 m above the apparatus, and videos were analyzed using Smart 3.0 software (Panlab Harvard Apparatus). The following variables were quantified: total distance traveled, bipedal positions, grooming, fecal granules, and time in zone (%); the central zone was defined as a 20 × 20 cm area [93,94,95].

#### 4.8.2. Elevated Plus Maze

Anxiolytic activity was evaluated using an elevated plus maze consisting of two open arms (30 × 5 cm), two closed arms (30 × 5×15 cm), and a central platform (5 × 5 cm), all constructed in a completely black finish and elevated 100 cm above the floor [96]. Thirty-five minutes after treatment administration, each mouse was placed on the central platform facing an open arm, monitored for 5 min under indirect lighting to minimize stress. Sessions were recorded using a Feeltek Elec HD Webcam (720p) positioned 1.50 m above the apparatus, and behavioral variables were analyzed with Smart 3.0 video-tracking software (Panlab Harvard Apparatus, Barcelona, Spain) [94,96].

#### 4.8.3. Rotarod Test

Motor coordination was evaluated using a rotarod apparatus consisting of a rotating bar 7 cm in diameter, subdivided into six compartments, positioned 25 cm above the floor and rotating at a constant speed of 12 rpm; the procedure followed the classical method [97]. Ten days prior to testing, all animals underwent an adaptation period during which they were trained on the rotarod to minimize novelty-induced stress. Only mice capable of remaining on the rotating bar for at least 2 min during the selection phase were included in the experiment. On the test day, animals received their assigned treatments: CMC 0.5% (control), diazepam 1 mg/kg diluted in CMC 0.5%, and hydroethanolic extract of *Schoenoplectus californicus* rhizomes at 50 or 200 mg/kg, both diluted in CMC 0.5% The rotarod test was performed 45 min after the administration of the respective treatments [97,98].

### 4.9. Analgesic Activity

Twenty-eight male Balb/c *Mus musculus* (45–55 g) were divided into four groups (*n* = 7): saline (10 mL/kg, oral, also used to resuspend both the extracts and drug), tramadol (Tramal^®^, Grünenthal; 5 mg/kg, oral, positive control), and hydroethanolic extract of *S. californicus* rhizomes (50 or 200 mg/kg, oral). Tramadol and the extract were diluted in sterile physiological saline solution to achieve their respective concentrations. All substances were administered via oral gavage.

#### 4.9.1. Analgesimeter Test

Mechanical pain threshold was assessed using the Randall-Selitto method with an Analgesy-Meter (Model 37215, Ugo Basile S.R.L., Gemonio, Italy) [99]. Pressure was applied to the hind paw via a blunt tip connected to a sliding counterweight system, measured in grams and converted to force units (N). The test ended upon observation of a nociceptive response (paw withdrawal, struggle, or vocalization) [99,100].

#### 4.9.2. Tail-Flick Test

Thermal nociception was evaluated using the D’Amour-Smith method [101]. The distal 4 cm of the tail was immersed in a 55 °C water bath, and latency to tail withdrawal was recorded, with a 25 s cutoff to prevent tissue damage [100,101].

#### 4.9.3. Hot Plate Test

The apparatus Hot Plate Analgesia Meter (Model 35100, Ugo Basile S.R.L., Gemonio, Italy) quantifies the specimen’s response threshold to a thermal stimulus. Supraspinal thermal nociception was measured at 55 °C. Mice were placed on the heated plate, and latency to hind-paw licking its paws or jumping was recorded, with a 25 s cutoff [100].

### 4.10. Procedure for In Silico Activity Prediction

Potential analgesic and sedative activities were predicted using the Prediction of Activity Spectra for Substances (PASS) system on the Way2Drug server. A total of 18 compounds—15 listed in Table 2 and three in Table 3—were selected for the in silico analysis, as these metabolites were identified through spectral matching and literature-based annotation, providing higher confidence in their structural assignment. Compounds annotated exclusively by SIRIUS and reported in Supplementary Table S2 were not included in this evaluation. Predictions in which the probability of activity (Pa) exceeded the probability of inactivity (Pi) were considered valid. Pa values were visualized in a heatmap using R (v4.3.3) [102,103].

### 4.11. Procedure for Physicochemical Predictions

Topological polar surface area (TPSA), consensus log P (CLogP), and P-glycoprotein (PGP) substrate status were predicted using SwissADME [40]. Gastrointestinal absorption and blood–brain barrier permeability were estimated using the BOILED-Egg model, visualized in R (v4.3.3) [101,103].

### 4.12. Experimental Design for Acute Oral Toxicity Testing

Acute oral toxicity was evaluated following the OECD Guideline 420 [104,105]. In accordance with the Fixed Dose Procedure, a sighting test was initially conducted at a dose of 300 mg/kg of the *S. californicus* hydroethanolic extract. This initial dose was administered to two fasting animals (one male and one female). Animals were fasted for 4 h prior to dosing and remained without food for 2 h post-administration, with water provided *ad libitum*. A minimum interval of 24 h was observed between dosing each sighting animal, as required by the guideline. Since no mortality or evident toxicity was observed at 300 mg/kg, the main study proceeded with the limit dose of 2000 mg/kg. Ten Balb/c mice (both sexes, *n* = 5 per group) were orally administered the extract. Animals were monitored for clinical signs of toxicity and mortality for the first 4 h post-administration and subsequently on a daily basis for 14 days. Individual body weights were recorded daily to detect any physiological variations. At the end of the study, mice were euthanized by cervical dislocation, and vital organs were collected and weighed for gross pathological examination. Detailed individual body weight data and daily monitoring are provided in Supplementary File S1 (Table S3). The comprehensive toxicity report, as originally recorded in Spanish, is available in Supplementary File S2.

### 4.13. Ethical Considerations

The study was approved by the Ethics Committee of the Faculty of Pharmacy and Biochemistry, National University of Trujillo (Approval No. IE-007-2018/CEFACFARM). Procedures adhered to the AVMA Guidelines for the Euthanasia of Animals (2020 Edition) (AVMA, 2020) and institutional animal care standards [105].

### 4.14. Statistical Analysis

Data are presented as mean ± standard error of the mean (SEM). Analyses were performed using SPSS v22.0, with one-way or two-way ANOVA followed by Tukey’s or Duncan’s multiple range (DMS) post hoc tests for significant differences. Graphs were generated with GraphPad Prism 7. Statistical significance was set at *p* < 0.05 [106,107,108].

## 5. Conclusions

This study provides a comprehensive evaluation of the hydroethanolic extract of *Schoenoplectus californicus* rhizomes, revealing its rich phytochemical profile, including flavonoids, phenolic compounds, and stilbenes, identified through LC-MS/MS and molecular networking. The extract demonstrated robust antioxidant capacity, significant anxiolytic and sedative effects in murine models, and notable analgesic activity, particularly at lower doses (50 mg/kg), comparable to standard drugs like diazepam and tramadol. The biphasic dose–response, with anxiolytic effects at moderate doses and sedation at higher doses, suggests a therapeutic window that requires optimization. In silico analyses support GABAergic modulation as a key mechanism, with several compounds predicted to cross the blood–brain barrier, enhancing their CNS activity. Collectively, these results validate the ethnopharmacological uses of *S. californicus* and position it as a promising candidate for developing natural therapeutic agents for anxiety and pain management. Future research should focus on elucidating specific receptor interactions, optimizing dosing regimens, and assessing bioavailability to advance its clinical potential.

## Figures and Tables

**Table 1:** Qualitative Phytochemical Screening of *Schoenoplectus californicus* Rhizome Extract.

Secondary Metabolite	Test	Result
Alkaloids	Dragendorff	−
	Wagner	−
	Mayer	−
Triterpenes and Steroids	Lieberman-Burchard	−
Flavonoids	Shinoda	+
Anthocyanins	Anthocyanidins	−
Lactones	Baljet	−
Phenolic Compounds	FeCl3	+++
Amino Acid and Amines	Ninhydrin	+
Quinones	Borntrager	−
Cardiotonic Glycosides	Kedde	−
Saponins	Foam	−
Tannins	Gelatin	++
Resins	Resins	−
Reducing Sugars	Fehling	+
Catechins	Catechins	−
Fats	Sudan	+
Polysaccharides	Mucilage	−

+: Positive; −: Negative. Intensity: + Low, ++ Moderate, +++ High.

**Table 2:** Features Annotated by Spectral Library Search Using GNPS Libraries.

Rt (min)	Observed *m/z*	Ion Identity	Compound Name	Molecular Formula (SIRIUS)	Weighted Cosine Similarity	Matching Signals	MSI Annotation Level
1.6	365.1055	[M + Na]^+^	Sucrose	C_12_H_22_O_11_	0.95	10/34	3
2.2	268.1041	[M + H]^+^	Adenosine	C_10_H_13_N_5_O_4_	0.88	17/44	3
2.2	294.1548	[M + H]^+^	N-fructosylisoleucine	C_12_H_23_NO_7_	0.85	30/57	2
2.2	229.1547	[M + H]^+^	N,N,N-trimethyl alanine-proline-betaine	C_21_H_20_N_2_O_3_	0.90	10/26	2
2.2	182.0813	[M + H]^+^	Tyrosine	C_9_H_11_NO_3_	0.96	22/52	2
3.9	205.0973	[M + H]^+^	Tryptophan	C_11_H_12_N_2_O_2_	0.88	18/26	2
7.9	291.0864	[M + H]^+^	Catechin	C_15_H_14_O_6_	0.83	13/52	3
10.3	198.1279	[M + H]^+^	dibenzylamine	C_14_H_15_N	0.698	6/6	2
11.2	197.081	[M + H]^+^	Xanthoxylin	C_10_H_12_O_4_	0.896	21/22	3
11.6	287.0551	[M + H]^+^	Kaempferol	C_15_H_10_O_6_	0.869	56/110	3
13.0	314.1387	[M + H]^+^	N-feruloyltyramine	C_18_H_19_NO_4_	0.796	9/21	2
13.4	301.1071	[M + H]^+^	Flavokawain C	C_17_H_16_O_5_	0.849	9/27	3
17.2	274.2739	[M + H]^+^	Lauryldiethanolamine	C_16_H_35_NO_2_	0.969	10/10	3
17.7	225.1961	[M + H]^+^	1,3-dicyclohexylurea	C_13_H_24_N_2_O	0.947	10/16	2
23.7	295.2268	[M + H]^+^	9-Oxo-octadecadienoic acid	C_18_H_30_O_3_	0.914	53/85	2

**Table 3:** UHPLC-HR-MS/MS characterization of major stilbenes detected in the hydroalcoholic extract of *S. californicus* rhizomes.

Rt (min)	Observed [M + H]^+^, *m*/*z*	Compound Name	Molecular Formula	Error (ppm)	MS/MS Fragments (Rel. Intensity %)	Reference
10.74	243.0665	Piceatannol	C_14_H_12_O_4_	1.0	243.0663 (100), 225.0556 (5), 201.0554 (22), 199.0763 (6), 175.0759 (6), 159.0445 (16)	[1]
11.84	485.1255	Scirpusin B	C_28_H_22_O_8_	2.7	485.1244 (100), 375.0872 (27), 362.0795 (13), 265.0508 (45), 109.0287 (24)	[2]
12.49	469.1298	Scirpusin A	C_28_H_22_O_7_	1.2	469.1295 (100), 385.1081 (7), 375.0879 (7), 359.0929 (7), 241.0506 (24), 227.0710 (7), 225.0557 (8), 213.0555 (8), 135.0444 (7), 109.0286 (11)	[1]

**Table 4:** Antioxidant Capacity of *Schoenoplectus californicus* Rhizome Hydroethanolic Extract (SCRHE).

Sample (cc mg/mL)		DPPH Assay		ABTS Assay	
		% Inhibition	IC_50_ (mg/mL)	% Inhibition	IC_50_ (mg/mL)
	1	65.3076 ± 0.7291	0.7319 ± 0.0098	72.5788 ± 1.0991	
SCRHE	0.1	16.4937 ± 0.7784		27.0189 ± 2.1991	0.6207 ± 0.0062
	0.01	6.7087 ± 0.1112		9.3962 ± 0.5698	
	0.001	5.6709 ± 0.3574		6.1887 ± 0.4713	
Trolox *			0.1793 ± 0.0038		0.1796 ± 0.0059

* Positive control. IC50: Half-maximal inhibitory concentration. Data are expressed as mean ± SEM (*n* = 3).

## Data Availability

The data presented in this study are available on request from the corresponding author.

## Supplementary Materials

The following supporting information can be downloaded at: https://www.mdpi.com/article/10.3390/md24050160/s1. File S1, including Figure S1: Molecular networking of *S. californicus* rhizomes (node sizes represent the sum of ion intensities); Figure S2: Molecular networking of major chemical classes in *S. californicus* rhizomes, showing class-specific subnetworks. Node sizes represent the sum of precursor ion intensities, and node color indicates the corresponding chemical class; Table S1: MZmine3 preprocessing parameters; Table S2: Molecules identified by SIRIUS analysis; Figure S3: MS/MS mirror plots of metabolites annotated in *S. californicus* via spectral library search using GNPS libraries; Table S3: Individual animal weight at day 0, day 7 and day 14 (acute toxicity); Figure S4: Representative locomotor trajectory plots obtained in the open field test for each experimental group: (a) CMC group (carboxymethylcellulose, 0.5%), (b) *Schoenoplectus californicus* (Totora) 50 mg/kg, (c) *Schoenoplectus californicus* (Totora) 200 mg/kg, and (d) diazepam (DZP, 1 mg/kg); Figure S5: Representative locomotor trajectory plots of exploratory behavior in the elevated plus maze test according to the experimental group: (a) CMC group (carboxymethylcellulose, 0.5%), (b) *Schoenoplectus californicus* (Totora) 50 mg/kg, (c) *Schoenoplectus californicus* (Totora) 200 mg/kg, and (d) diazepam (DZP, 1 mg/kg); and Figure S6: Taxonomic identification of *S. californicus* at *Herbarium Truxillense* (HUT). File S2: Comprehensive Acute Oral Toxicity report in *S. californicus* (original Spanish version).

## Abbreviations

The following abbreviations are used in this manuscript:

ABTS	2,2′-Azinobis(3-ethylbenzothiazoline-6-sulfonic acid)
BBB	Blood–brain barrier
CNS	Central nervous system
CMC	Carboxymethylcellulose
DPPH	2,2-Diphenyl-1-picrylhydrazyl
EPM	Elevated plus maze
HESI	Heated electrospray ionization
IC_50_	Half maximal inhibitory concentration
LC–MS/MS	Liquid chromatography–tandem mass spectrometry
LD_50_	Median lethal dose
MS	Mass spectrometry
NCE	Normalized collision energy
OECD	Organisation for Economic Co-operation and Development
PASS	Prediction of Activity Spectra for Substances
PGP	P-glycoprotein
SEM	Standard error of the mean
TPSA	Topological polar surface area
UHPLC	Ultra-high-performance liquid chromatography
