# Supplementary material for: Schoenoplectus californicus (C.A. Mey.) Soják: Chemical Profile, Antioxidant Capacity, Psychopharmacological Exploration and Analgesic Activity
Source: Mar Drugs. 2026 Apr 30;24(5):160. doi: 10.3390/md24050160 (PMC13208810; doi:10.3390/md24050160)
Supplement: Supplementary file 1 [file marinedrugs-24-00160-s001.zip › Figure S2_IC.pptx]

## Slide 1
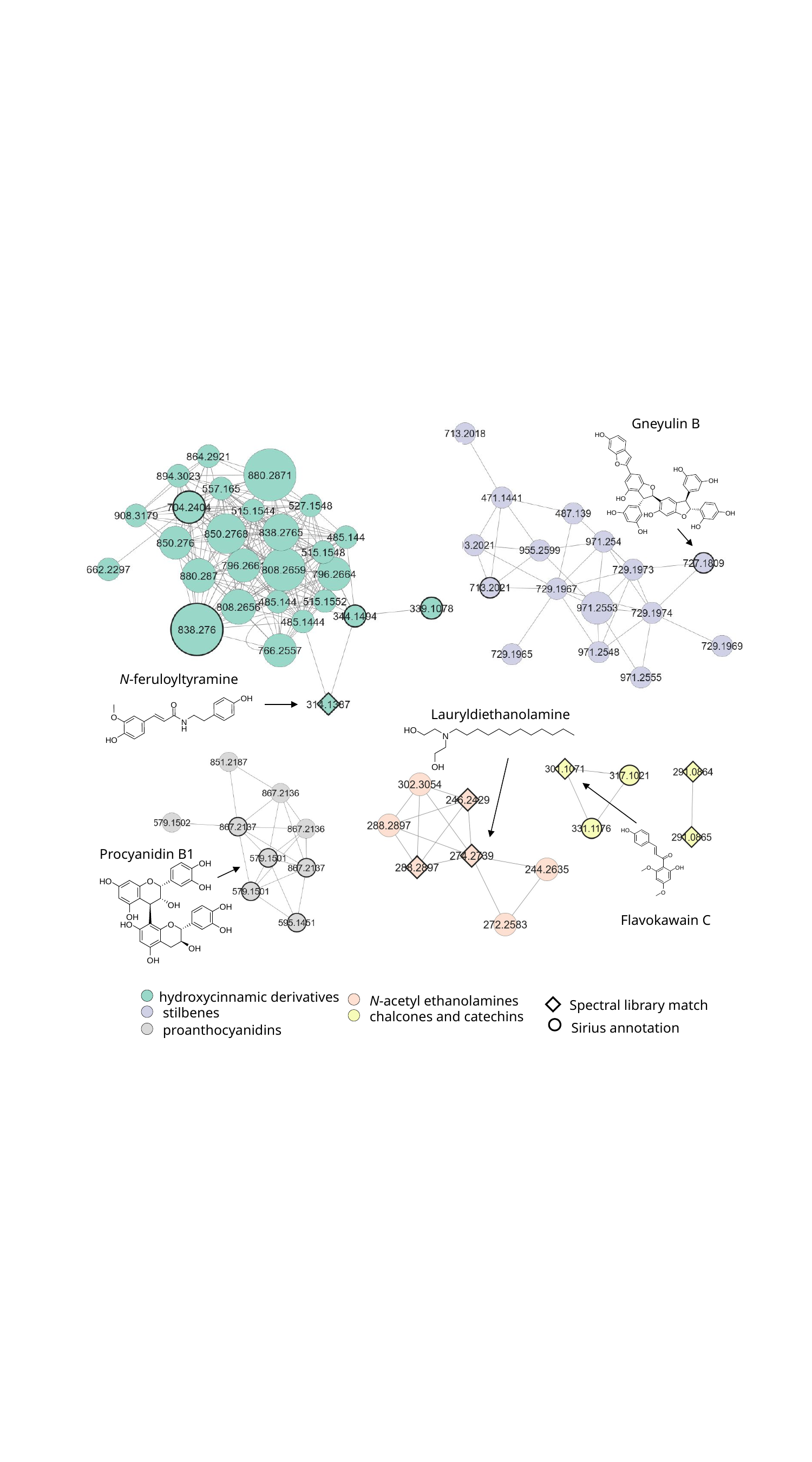

Gneyulin B
N-feruloyltyramine
Lauryldiethanolamine
Procyanidin B1
Flavokawain C
Spectral library match
hydroxycinnamic derivatives
 N-acetyl ethanolamines
Sirius annotation
 stilbenes
 chalcones and catechins
 proanthocyanidins
