# Supplementary material for: Schoenoplectus californicus (C.A. Mey.) Soják: Chemical Profile, Antioxidant Capacity, Psychopharmacological Exploration and Analgesic Activity
Source: Mar Drugs. 2026 Apr 30;24(5):160. doi: 10.3390/md24050160 (PMC13208810; doi:10.3390/md24050160)
Supplement: Supplementary file 1 [file marinedrugs-24-00160-s001.zip › Supplementary file S1.pdf]

Supplementary file S1

Schoenoplectus californicus (C.A. Mey.) Soják: Chemical profile, antioxidant capacity, psychopharmacological exploration and analgesic activity

Julio Campos-Florián[1*], Gladys Galliani-Huamanchumo[2], Alessandra Campos-Bazán[2], Betsabé Chunga-Flores[1], Inés Castro-Dionicio[3], Víctor Villarreal-La Torre[1], Lucia Flores-Atoche[2], Lucia Gonzales-Mendez[2], Gianfranco Ramos-Farfán[2], José Condor-Goytizolo[2], Ana Guevara-Vásquez[1], Marilú Soto-Vásquez[4], Juan Rodríguez-Soto[5], Paul Alvarado-García[6], William Sagástegui-Guarniz and Billy Cabanillas-Amado[6]

[1] Department of Pharmacology, Faculty of Pharmacy and Biochemistry, Universidad Nacional de Trujillo, 13011 Trujillo, Peru; jcamposf@unitru.edu.pe (J.C.-F.); bchugaf@unitru.edu.pe (B.Ch.-F.); vvillarreal@unitru.edu.pe (V.V.-L.T.); aguevara@unitru.edu.pe (A.G.-V); wsagasteguig@unitru.edu.pe (W.S.-G.)

[2] Faculty of Pharmacy and Biochemistry, Universidad Nacional de Trujillo, 13011 Trujillo, Peru; ggalliani@unitru.edu.pe (G.G.-H.); acamposb@unitru.edu.pe (A.C.-B.); lfloresa@unitru.edu.pe (L.F.-A.); lgonzalesm@unitru.edu.pe (L.G.-M); jcondor@unitru.edu.pe (J.C.-G)

[3] EGENODIA Inserm U1283, CNRS UMR8199, Institute Pasteur de Lille, Lille University Hospital, Université de Lille, 59045, Lille, France; ines.castro@univ-lille.fr (I.C.-D.)

[4] Department of Pharmacotechnics, Faculty of Pharmacy and Biochemistry, Universidad Nacional de Trujillo, 13011 Trujillo, Peru; msoto@unitru.edu.pe (M.S.-V.)

[5] Biological Sciences Faculty, Universidad Nacional de Trujillo, 13011 Trujillo, Peru; jrodrigezs@unitru.edu.pe (J.R.-S.)

[6] Universidad Autónoma del Perú, 150142 Lima, Perú; palvaradog@autonoma.edu.pe (P.A-G.)

[7] Natural Products Research Unit, Research and Development Laboratories, Faculty of Sciences and Philosophy, Universidad Peruana Cayetano Heredia, Lima, Peru; billy.cabanillas.a@upch.pe (B.C.-A.)

* Correspondence: jcamposf@unitru.edu.pe; Tel.: +51 971 854 612

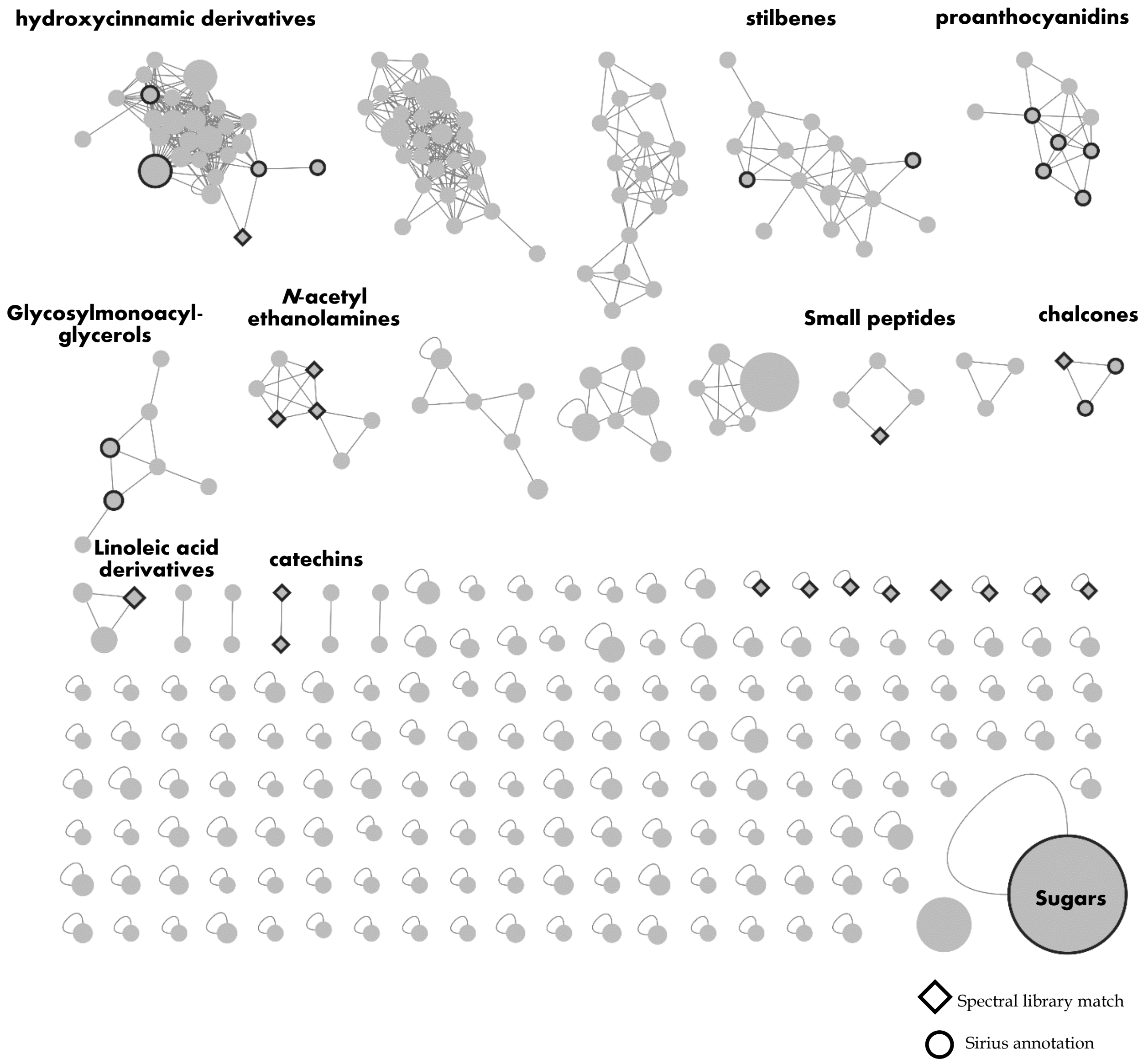

Figure S1. Molecular networking of *S. californicus* rhizomes (node sizes represent the sum of ion intensities).

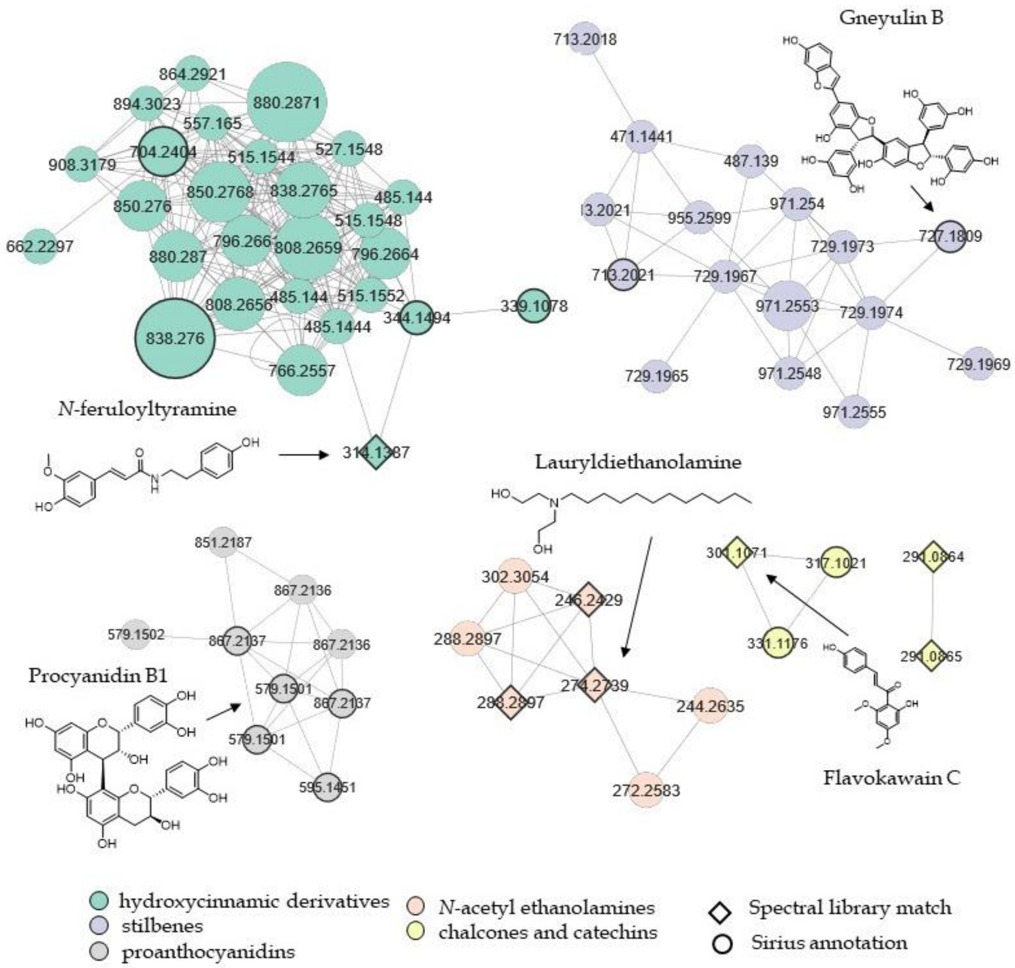

Figure S2. Molecular networking of major chemical classes in *S. californicus* rhizomes, showing class-specific subnetworks. Node sizes represent the sum of precursor ion intensities, and node color indicates the corresponding chemical class

Table S1. MZmine3 preprocessing parameters

**Mass detection**	
Noise level: MS level 1	100000
m/z tolerance: MS level 1	0.0015 *m/z* or 5.0 ppm
Noise level: MS level 2	10
m/z tolerance: MS level 2	0.0015 *m/z* or 5.0 ppm
**ADAP Chromatogram Builder**	
Minimum consecutive scans	3
Minimum intensity for consecutive scans	100000
Minimum absolute height	100000
m/z tolerance (scan-to-scan)	0.0015 *m/z* or 5.0 ppm
**Local minimum resolver**	
MS1 to MS2 precursor tolerance	0.0015 *m/z* or 5.0 ppm
Chromatographic threshold	0.85
Minimum absolute height	100000.0
Min ratio of peak top/edge	1.7
Peak duration range (min)	1.0
Minimum scans (data points)	3
**13C isotope filter**	
m/z tolerance (intra-sample)	0.0015 *m/z* or 5.0 ppm
Retention time tolerance (min)	0.03
Representative isotope	true
**Join aligner**	
m/z tolerance (sample-to-sample)	0.0015 *m/z* or 5.0 ppm
Weight for m/z	1.0
Retention time tolerance (min)	0.5
Weight for RT	1.0
**Filtering**	
Minimum aligned features (samples)	2
**Blank subtraction**	
Quantification	Height
Ratio type	MAXIMUM
Fold change increase	3.0
Keep or remove features below fold change	Remove - Only keep features above fold change
**Correlation grouping**	
Retention time tolerance (min)	0.1
Minimum feature height	100000
Intensity threshold for correlation:	100000
Min feature shape correlation (Pearson)	0.85
Min feature height correlation (Pearson)	0.70
**Ion identity networking**	

m/z tolerance (intra-sample)	0.0015 *m/z* or 5.0 ppm
Maximum charge	2
Maximum molecules/cluster	3
Adducts	$[M+H]^{+,}$ $[M+Na]^{+}$, $[M+K]^{+}$, $[M+NH_4]^{+}$ /$[M-H]^{-}$
Modifications	$[M-H_2O]$, $[M-H_2O]$/ $[M+HFA]$

Table S2. Molecules identified by SIRIUS analysis, complementing spectral library-based identifications.

Feature	MZmine id	Area	RT	m/z	Height	Ion identities	Spectral db matches (GNPS library):					SIRIUS:			MSI annotation level
							Compound name	Mol formula	Precursor mz	Cosine score	N matching signals	mol formula	CSI-Finger ID	Canopus	
23	500	1.16E+07	1.6	365.1055	8.37E+07	[M+Na]+	Sucrose	C12H22O11	365.105	0.95	Oct-34	C12H22O11	Sucrose	Disaccharides	3
4	670	4.23E+05	2.2	268.10409	4.27E+06	[M+H]+	Adenosine	C10H13N5O4	268.104	0.88	17/44	C10H13N5O4	Adenosine	Purine nucleosides	3
14	672	7.19E+05	2.2	294.15475	9.24E+06	[M+H]+	N-Fructosyl isoleucine	C12H23NO7	294.156	0.85	30/57	C12H23NO7	N-Fructosyl isoleucine	Small peptides	2
5	684	1.83E+05	2.2	229.15474	2.83E+06	[M+H]+	N,N,N-trimethyl-l-alanine-l-proline betaine	C21H20N2O3	229.155	0.9	26-Oct	C21H20N2O3	Isoleucylproline	Proline and derivatives	2
12	683	2.90E+05	2.2	182.08125	3.77E+06	[M+H]+	Tyrosine	C9H11NO3	182.08	0.96	22/52	C9H11NO3	Tyrosine	Phenylalanine and derivatives	2
15	697	1.59E+05	3.9	205.0973	7.67E+05	[M+H]+	Tryptophan	C11H12N2O2	205.097	0.88	18/26	C11H12N2O2	L-Tryptophan	Indoles	2
6	698	1.70E+05	4.3	579.15009	5.48E+05	[M+H]+	-	-	-	-	-	C30H26O12	Procyanidin B1	Proanthocyanidins	3
7	699	1.56E+05	4.5	595.14513	4.16E+05	[M+H]+	-	-	-	-	-	C30H26O13	Procyanidin	Proanthocyanidins	3
8	703	4.25E+05	5.7	867.21366	8.45E+05	[M+H]+	-	-	-	-	-	C45H38O18	Arecatannin B1	Proanthocyanidins	3
27	704	1.75E+05	5.9	291.08646	6.82E+05	[M+H]+	-	-	-	-	-	C15H14O6	Catechin	Catechins	3
9	709	3.91E+05	7	579.1501	9.95E+05	[M+H]+	-	-	-	-	-	C30H26O12	Procyanidin B1	Proanthocyanidins	3

28	716	3.38E+04	7.9	291.086642	3.28E+05	[M+H]+	Catechin	C15H14O6	291.087	0.83	13/52	C15H14O6	Catechin	Catechins	3
10	725	1.43E+05	8.4	867.21372	4.80E+05	[M+H]+	-	-	-	-	-	C45H38O18	Proanthocyanidin C1	Proanthocyanidins	3
24	743	3.12E+04	10	339.10783	3.38E+05	[M+H]+	-	-	-	-	-	C16H18O8	4-methylumbelliferyl glucoside	Coumarins	3
11	755	2.40E+05	10.3	198.12789	3.93E+05	[M+H]+	Dibenzylamine	C14H15N	198.128	0.7	6-Jun	C14H15N	Bibenzylamine	Phenylmethylamines	2
29	782	3.41E+04	11.2	197.08101	3.53E+05	[M+H]+	2-Hydroxy-4,6-dimethoxyacetophenone	C10H12O4	197.081	0.9	21/22	C10H12O4	Xanthoxylin	Alkyl-phenylketones	3
22	789	8.77E+05	11.6	287.05506	2.04E+06	[M+H]+	Kaempeferol	C15H10O6	287.055	0.87	56/110	C15H10O6	Kaempferol	Flavonols	3
25	808	1.79E+06	12	317.1021	4.30E+06	[M+H]+	Sulfadimethoxine-d6*	C12H14N4O4S*	317.118*	0.73*	8/45*	C17H16O6*	3-Methoxyhelichrysetin	Chalcones	3
2	863	2.05E+05	12.7	727.18093	1.19E+06	[M+H]+	-	-	-	-	-	C42H30O12	Gneyulin B	Stilbene trimers	3
16	880	1.33E+05	13	314.13872	1.20E+06	[M+H]+	N-feruloyltyramine	C18H19NO4	314.139	0.8	21-Sep	C18H19NO4	Moupinamide	Hydroxycinnamic acid and derivatives	3
17	893	8.91E+04	13.1	704.24037	9.32E+05	[M+NH4]+	-	-	-	-	-	C30H38O18	1-O-Acetyl-6-O-(3-methoxy-4-hydroxy-trans-cinnamoyl)-beta-D-fructofuranosyl 2-O,4-O,6-O-triacetyl-alpha-D-glucopyranoside	Hydroxycinnamic acid and derivatives	3
18	901	5.02E+04	13.3	344.14936	4.84E+05	[M+H]+	-	-	-	-	-	C19H21NO5	N-trans-Feruloyl-3-methoxytyramine (N-Feruloyl-3-O-	Hydroxycinnamic acid and derivatives	3

													methyldopamine)		
30	905	3.83E+05	13.4	301.10713	3.63E+06	[M+H]+	Flavokawain C	C17H16O5	301.107	0.85	27-Sep	C17H16O5	Flavokawain C	Chalcones	3
3	916	5.02E+05	13.7	713.20215	3.66E+06	[M+H]+	-	-	-	-	-	C42H32O11	4-[3-(3,5-dihydroxyphenyl)-7-[3-(3,5-dihydroxyphenyl)-6-hydroxy-4-[2-(4-hydroxyphenyl) ethenyl]-2,3-dihydro-1-benzofuran-2-yl]-4-hydroxy-2,3-dihydro-1-benzofuran-2-yl]benzene-1,3-diol	Stilbene	3
26	918	2.89E+05	13.8	331.11765	2.32E+06	[M+H]+	"(2E)-3-(4,7-dimethoxy(2H-benzo[d]1,3-dioxolen-5-yl))-1-(2-fluorophenyl) prop-2- en-1-one*	C18H15FO5*	331.098*	0.77*	17/82*	C18H18O6*	2',4-dihydroxy-3,4',6'-trimethoxychalcone	Chalcones	3
31	935	2.53E+04	14.6	246.242429	3.48E+05	[M+H]+	-	-	-	-	-	C16H35NO2	Ethanol, 2,2'-(decylimino)bis-	1,2-aminoalcohols	3
19	962	2.41E+06	15.2	838.27596	1.89E+07	[M+NH4]+	-	-	-	-	-	C38H44O20	1-O-Acetyl-3-O,6-O-bis(3-methoxy-4-hydroxy-trans-cinnamoyl)-beta-D-fructofuranosyl 2-O,6-O-diacetyl-alpha-D-glucopyranoside	Hydroxycinnamic acid and derivatives	3
32	1026	2.16E+06	17.2	274.27391	2.19E+07	[M+H]+	Lauryldiethanolamine	C16H35NO2	274.274	0.97	10-Oct	C16H35NO2	N-Lauryldiethanolamine	1,2-aminoalcohols	3
1	1043	4.68E+06	17.7	225.1	1.96E+07	[M+H]+	1,3-Dicyclohexylurea	C13H24N2O	225.196	0.95	16-Oct	C13H24N2O	1,3-Dicyclohexylurea	Ureas	2

				9612											
33	1058	1.65E+05	18	288.28968	1.33E+06	[M+H]+	-	-	-	-	-	$C_{16}H_{35}NO_2$	2,2'-(tridecylimino) diethanol	1,2-aminoalcohols	3
20	1178	3.11E+05	21.6	672.41673	2.88E+06	[M+NH4]+	-	-	-	-	-	$C_{31}H_{58}O_{14}$	2-hydroxy-3-{[3,4,5-trihydroxy-6-({[3,4,5-trihydroxy-6-(hydroxymethyl)oxan-2-yl]oxy}methyl) oxan-2-yl]oxy}propyl hexadecanoate	Glycosylmonoacyl-glycerols	3
21	1209	2.31E+05	22.3	698.43237	2.27E+06	[M+NH4]+	-	-	-	-	-	$C_{33}H_{60}O_{14}$	Gingerglycolipid A; 12',13',15',16'-Tetrahydro	Glycosylmonoacyl-glycerols	3
13	1324	1.64E+05	23.7	295.22677	2.06E+06	[M+H]+	9-Oxo-octadecadienoic acid	$C_{18}H_{30}O_3$	295.226	0.91	53/85	$C_{18}H_{30}O_3$	13-Oxo-9,11-Octadecadienoic Acid	Linoleic acid derivatives	2

*For these features, SIRIUS annotation was prioritized over the spectral library match

Sucrose

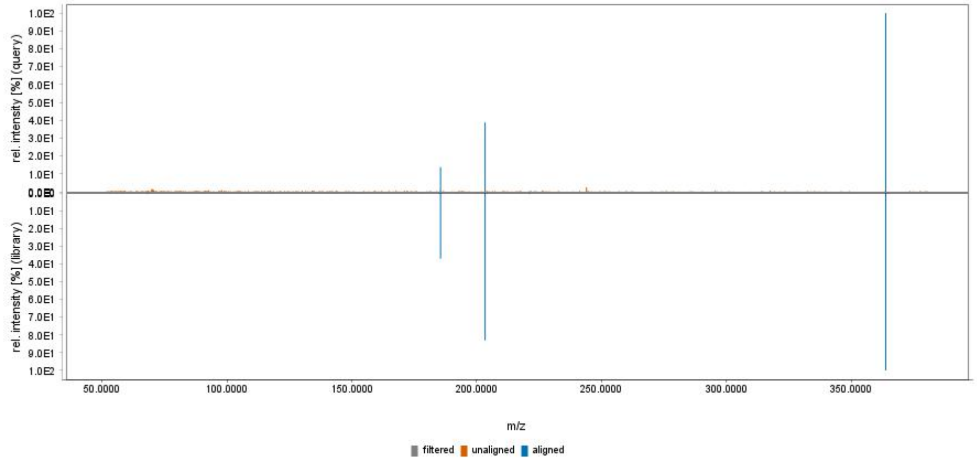

Adenosine

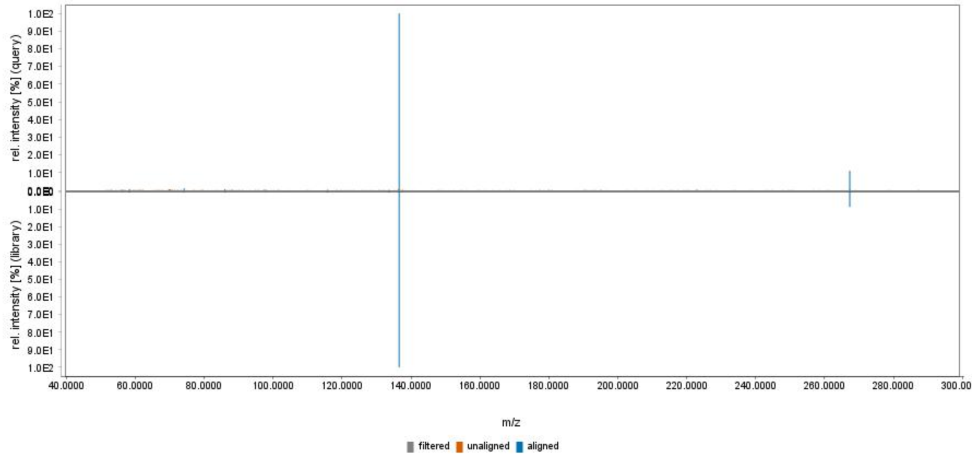

N-fructosylisoleucine

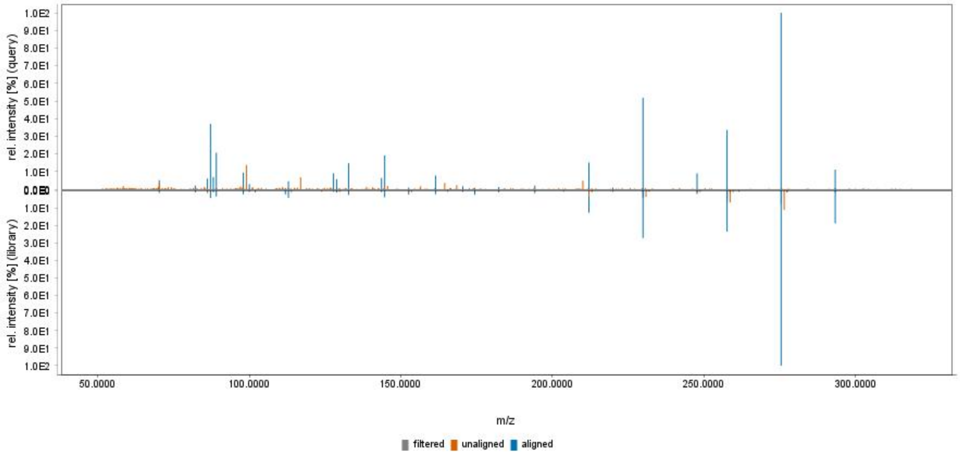

Figure S3. MS/MS mirror plots of metabolites annotated in *S. californicus* via spectral library search using GNPS libraries.

N,N,N-trimethyl-l-alanine-l-proline betaine

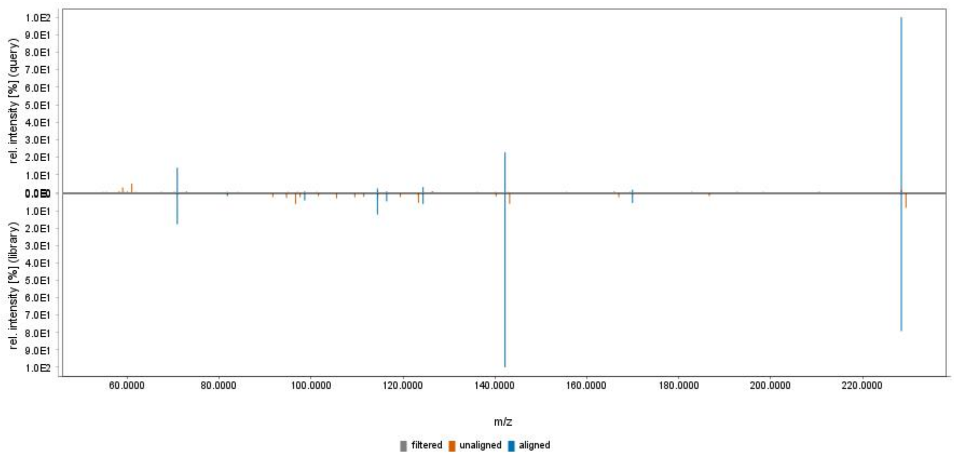

Tyrosine

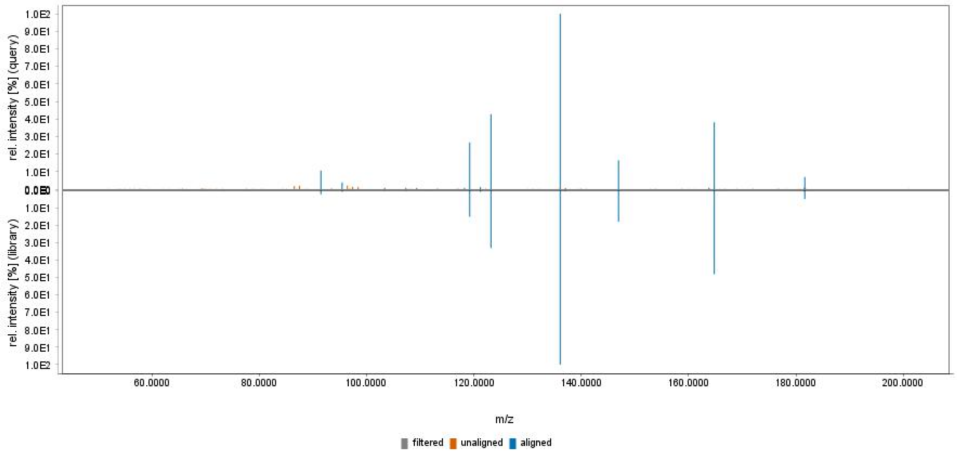

Tryptophan

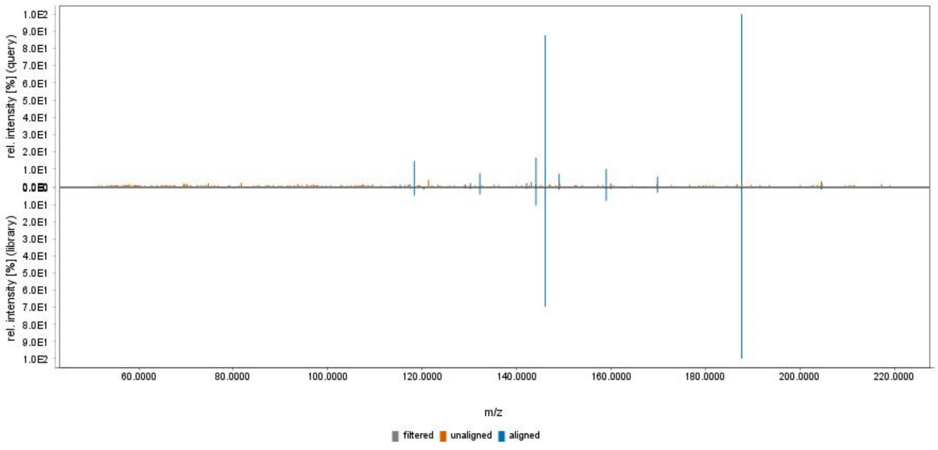

Figure S3 (continued)

Catechin

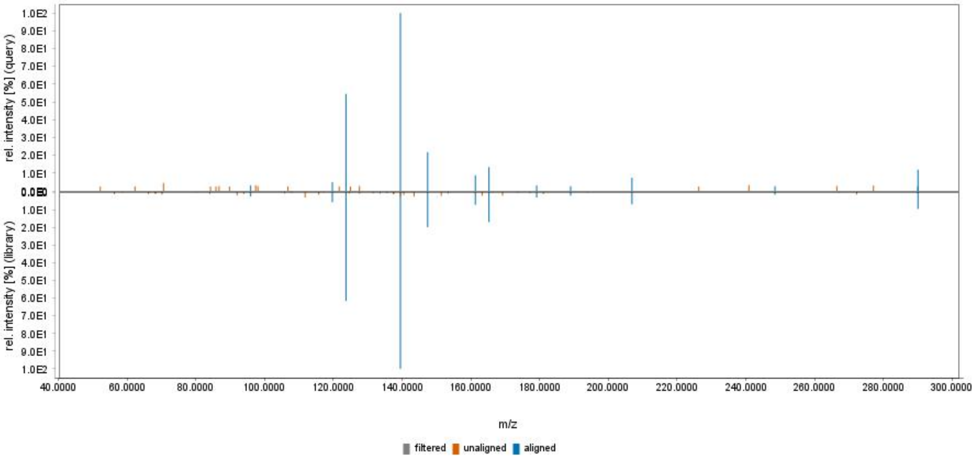

Dibenzylamine

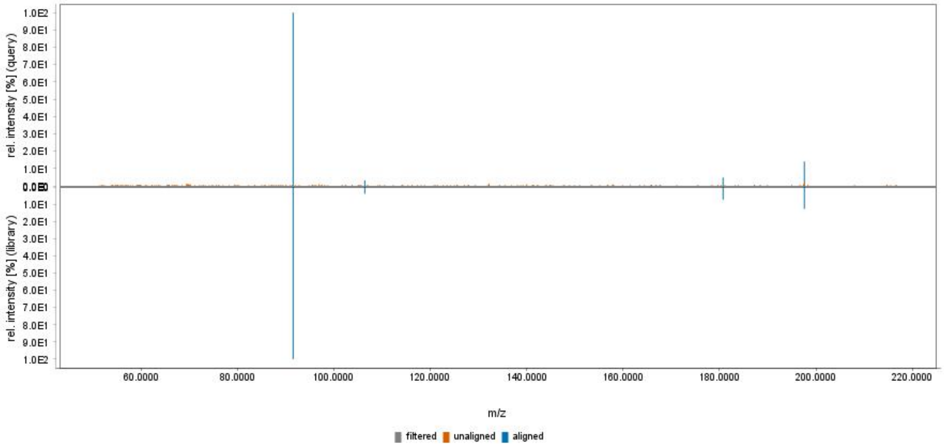

Xanthoxylin

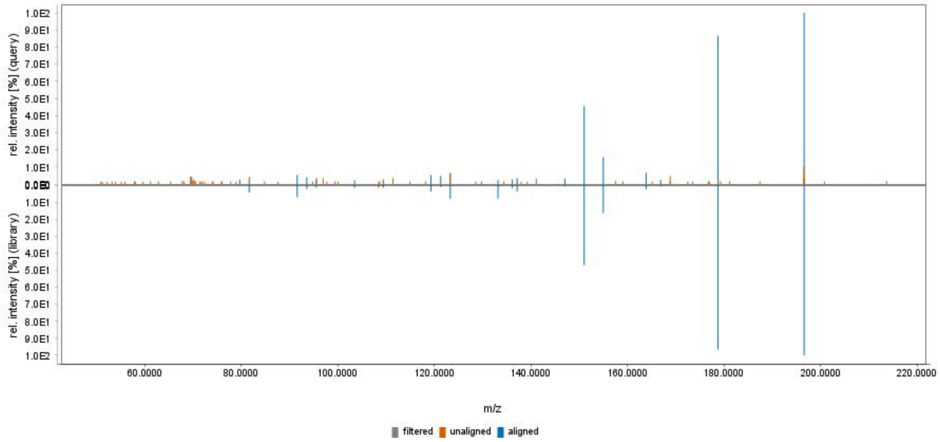

Figure S3 (continued).

Kaempferol

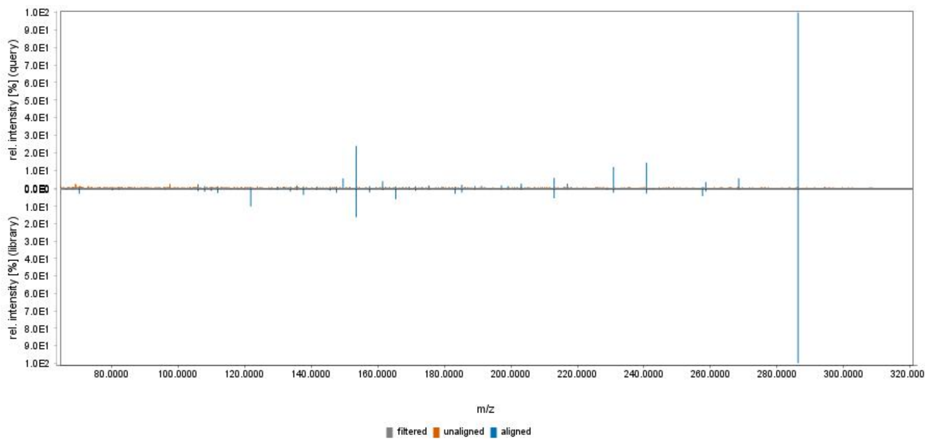

N-feruloyltyramine

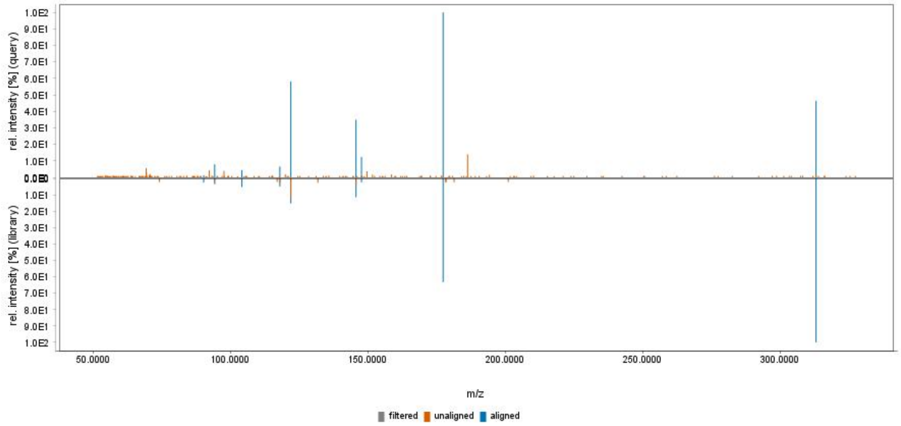

Flavokawain C

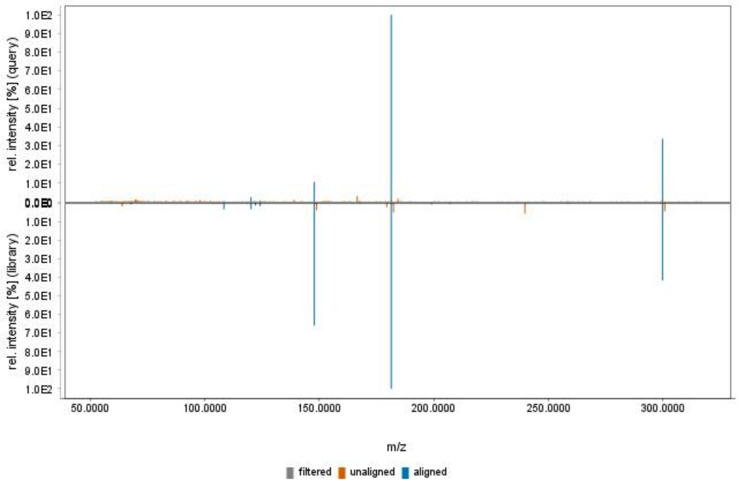

Figure S3 (continued).

Lauryldiethanolamine

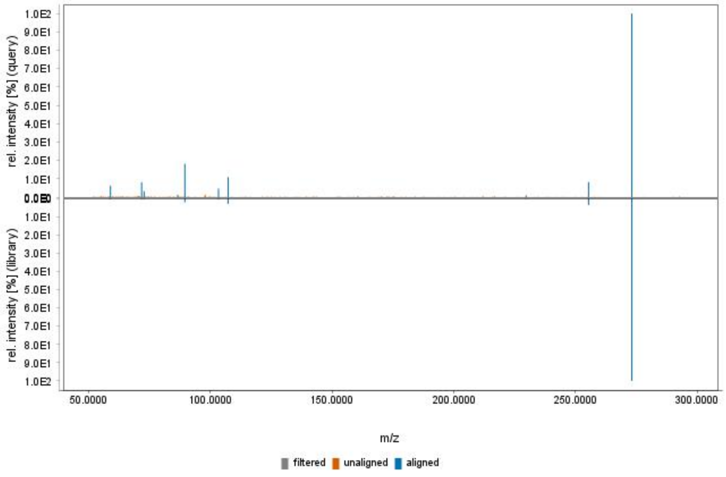

1,3-dicyclohexylurea

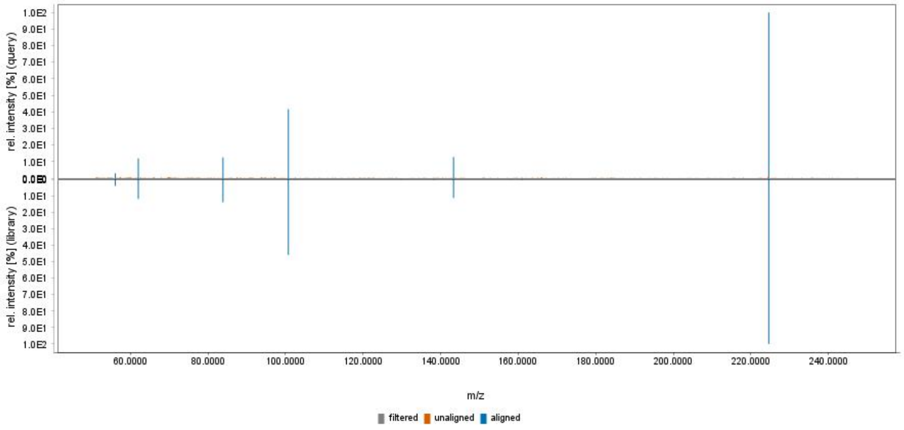

9-Oxo-octadecadienoic acid

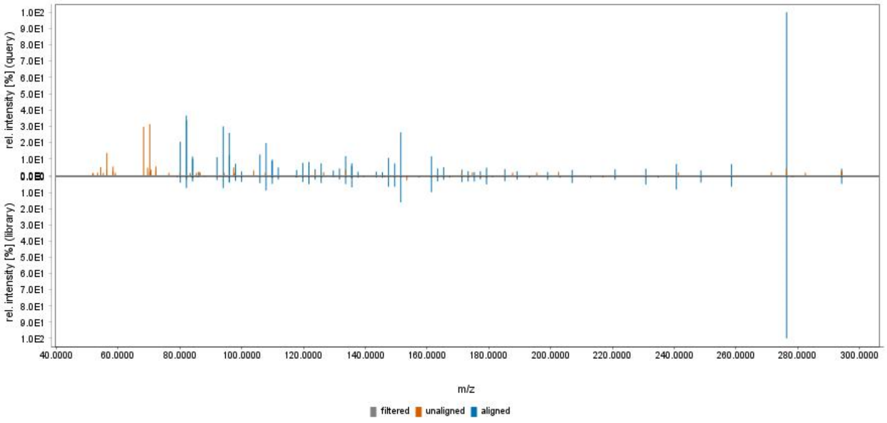

Figure S3 (continued).

Table S3. Individual animal weight at day 0, day 7 and day 14 (acute toxicity)

	Animal weight (g)		
	Day 1	Day 7	Day 14
M1	33	+	+
M2	32	39	36
M3	39	39	35
M4	46	48	44
M5	35	27	24
F1	44	43	42
F2	38	40	39
F3	37	+	+
F4	41	41	39
F5	46	40	36

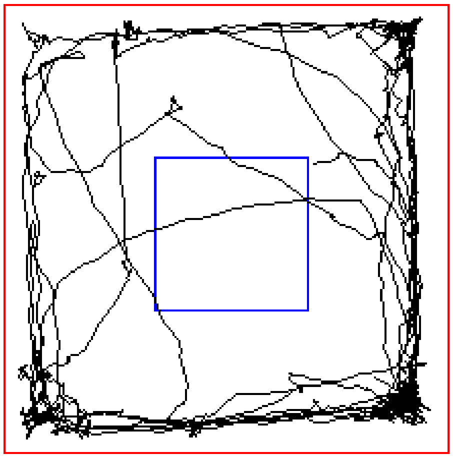

(a)

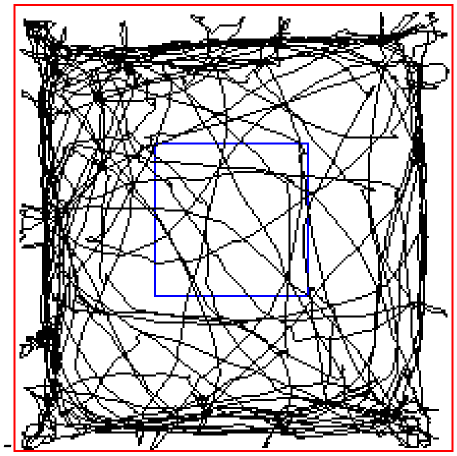

(b)

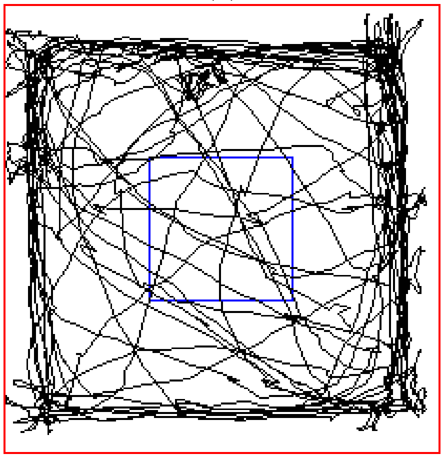

(c)

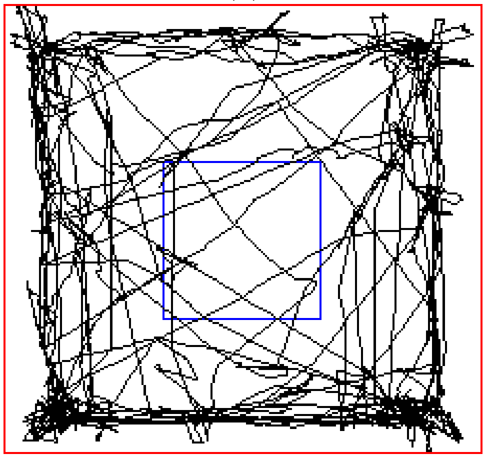

(d)

Figure S4. Representative locomotor trajectory plots obtained in the Open Field Test for each experimental group: (a) CMC group (carboxymethylcellulose, 0.5%), (b) *Schoenoplectus californicus* (Totora) 50 mg/kg, (c) *Schoenoplectus californicus* (Totora) 200 mg/kg, and (d) diazepam (DZP, 1 mg/kg).

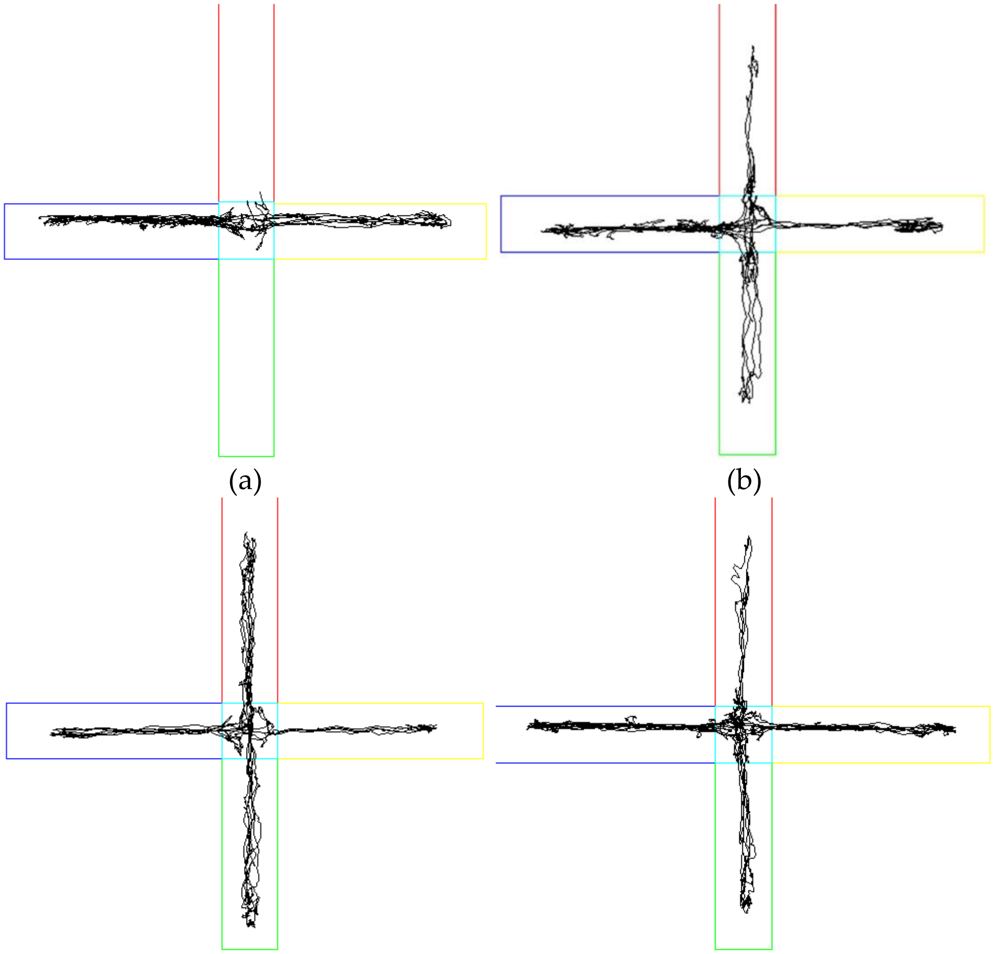

(a) (b) (c) (d)

Figure S5. Representative locomotor trajectory plots of exploratory behavior in the Elevated Plus Maze Test according to the experimental group: (a) CMC group (carboxymethylcellulose, 0.5%), (b) *Schoenoplectus californicus* (Totora) 50 mg/kg, (c) *Schoenoplectus californicus* (Totora) 200 mg/kg, and (d) diazepam (DZP, 1 mg/kg).

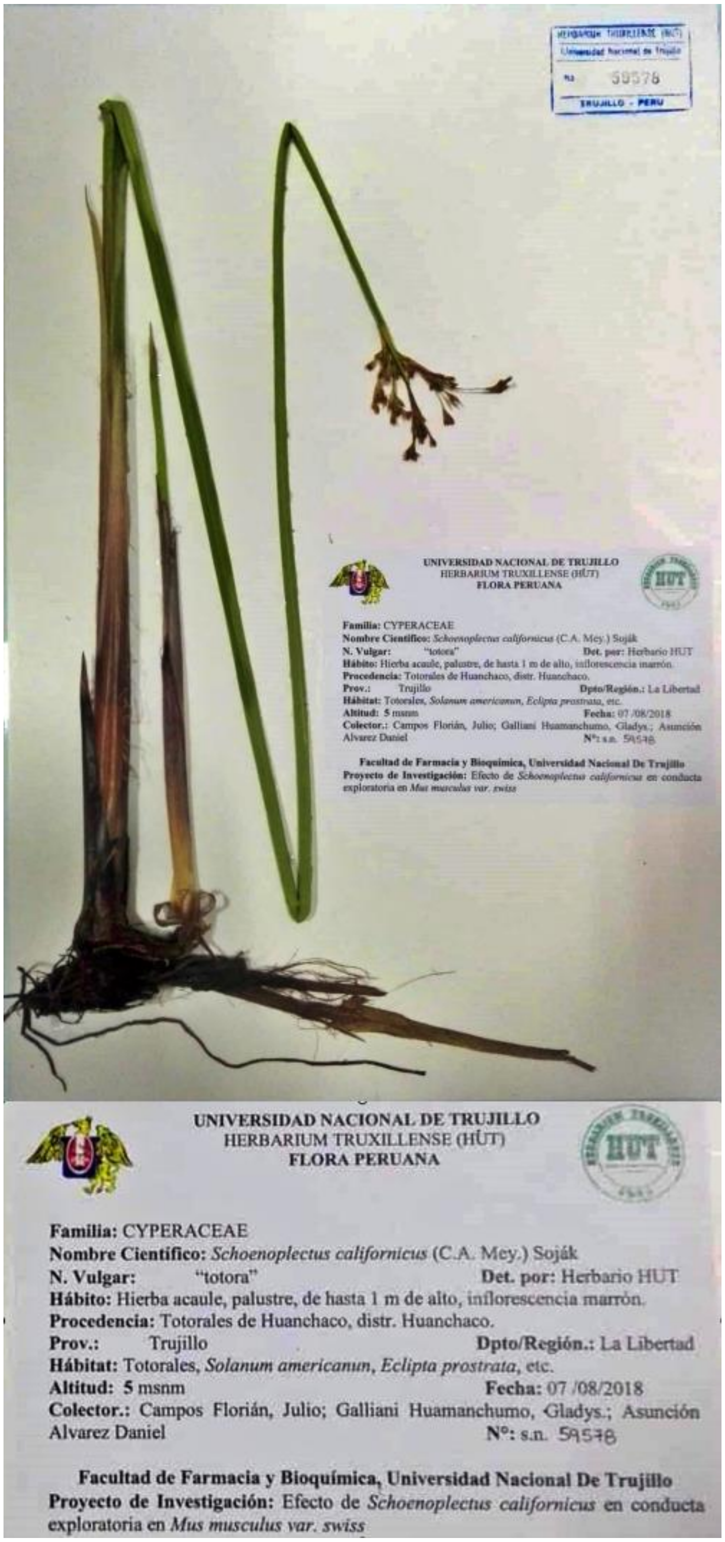

Figure S6. Taxonomic identification of *S. californicus* in to *Herbarium Truxillense* (HUT)
