# Supplementary material for: Schoenoplectus californicus (C.A. Mey.) Soják: Chemical Profile, Antioxidant Capacity, Psychopharmacological Exploration and Analgesic Activity
Source: Mar Drugs. 2026 Apr 30;24(5):160. doi: 10.3390/md24050160 (PMC13208810; doi:10.3390/md24050160)
Supplement: Supplementary file 1 [file marinedrugs-24-00160-s001.zip › Supplementary File S2_english (AcuteToxicity).pdf]

Supplementary File S2

GROUP TOXICITY REPORT

**Title:**	*Acute Oral toxicity*				
**Substance tested:**	Hydroethanolic extract 40° Totora	**Purity:**	Et. OH 50° (5% w/v)	**Vehicle:**	Physiological Saline Solution 0.9% (SSF)
**Specimen:**	**Age:**	**Sex:**	**Origin:**	**Diet:**	**Accommodation:**
Mus musculus Balb/c	6 months	Female Male	INS Chorrillos	Conejina	Bioterium Faculty FF.BB
**Experimental Group:**		Group II		**Dosage:**	2000 mg/kg

Evolution of body weight

**Specimen**	**Body weight**			
	**Day 1**	**Day 7**	**Day 14**	**Δ Weight**
**1**	33 g	+	+	-
**2**	32 g	39 g	36 g	4 g
**3**	39 g	39 g	35 g	-4 g
**4**	46 g	48 g	44 g	-2 g
**5**	35 g	27 g	24 g	-11 g
**6**	44 g	43 g	42 g	-2 g
**7**	38 g	40 g	39 g	1 g
**8**	37 g	+	+	-
**9**	41 g	41 g	39 g	-2 g
**10**	46 g	40 g	36 g	-10 g

Evaluation of signs and symptoms

Dosage (mg/kg)	Specimen Code	Effects After Dosing (Hours)								Effects After Dosage (Days)													
		½	1	2	3	4	10	12	18	1	2	3	4	5	6	7	8	9	10	11	12	13	14
2000 mg/kg	**R1-2000F**	Pil.<br>Sle.<br>Tach.<br>Trem.	Pil.<br>Sle.<br>Tach.<br>Trem..	Pil.<br>Sle.<br>Tach.<br>Trem.	Pil.<br>Sle.<br>Tach.<br>Trem.	Pil.<br>Sle.<br>Tach.<br>Trem.	Pil.<br>Sle.<br>Tach.<br>Trem.	Pil.<br>Sle.<br>Tach.<br>Trem.	Pil.<br>Sle.<br>Tach.<br>Trem.	+													
	**R2-2000F**	Pil.<br>Sle.<br>Tach.<br>Trem.	Pil.<br>Sle.<br>Tach.<br>Trem.	Pil.<br>Sle.<br>Tach.<br>Trem.	Pil.<br>Sle.<br>Tach.<br>Trem..	Pil.<br>Sle.<br>Tach.<br>Trem.	Pil.<br>Sle.<br>Tach.<br>Trem.	Pil.<br>Sle.<br>Tach.<br>Trem.	Pil.<br>Sle.<br>Tach.<br>Trem.	Pil.<br>Sle.<br>Tach.<br>Trem.	Pil.	-											
	**R3-2000M**	Pil.<br>Tach.<br>Trem.	Pil.<br>Tach.<br>Trem.	Pil.<br>Sle.<br>Tach.<br>Trem.	Pil.<br>Sle.<br>Tach.<br>Trem.	Pil.<br>Sle.<br>Tach.<br>Trem.	Pil.<br>Sle.<br>Tach.	Pil.<br>Sle.	Pil.<br>Sle.	Pil.	Pil.	-											
	**R4-2000M**	Pil.<br>Tach.<br>Trem.	Pil.<br>Tach.<br>Trem.	Pil.<br>Tach.<br>Trem.	Pil.<br>Sle.<br>Tach.<br>Trem.	Pil.<br>Sle.<br>Tach.<br>Trem.	Pil.<br>Sle.<br>Tach.	Pil.<br>Sle.	Pil.<br>Sle.	Pil.	Pil.	-											
	**R5-2000F**	Pil.<br>Sle.<br>Tach.<br>Trem.	Pil.<br>Sle.<br>Tach.<br>Trem.	Pil.<br>Sle.<br>Tach.<br>Trem.	Pil.<br>Sle.<br>Tach.<br>Trem.	Pil.<br>Sle.<br>Tach.<br>Trem.	Pil.<br>Sle.<br>Tach.	Pil.<br>Sle.<br>Tach.	Pil.<br>Sle.<br>Tach.	Pil.<br>Sle.	Pil.	-											
	**R6-2000F**	Pil.<br>Sle.<br>Tach.<br>Trem.	Pil.<br>Sle.<br>Tach.<br>Trem.	Pil.<br>Sle.<br>Tach.<br>Trem.	Pil.<br>Sle.<br>Tach.<br>Trem.	Pil.<br>Sle.<br>Tach.<br>Trem.	Pil.<br>Sle.<br>Tach.<br>Trem.	Pil.<br>Sle.<br>Tach.<br>Trem.	Pil.<br>Sle.<br>Tach.<br>Trem.	Pil.<br>Sle.<br>Tach.	Pil.	-											
	**R7-2000M**	Pil.<br>Sle.<br>Tach.<br>Trem.	Pil.<br>Sle.<br>Tach.<br>Trem.	Pil.<br>Sle.<br>Tach.	Pil.<br>Sle.<br>Tach.<br>Trem.	Pil.<br>Sle.<br>Tach.<br>Trem.	Pil.<br>Sle.<br>Tach.	Pil.<br>Sle.<br>Tach.	Pil.<br>Sle.<br>Tach.	Pil.<br>Sle.<br>Tach.	Pil.	-											
	**R8-2000M**	Pil.<br>Sle.<br>Tach.	Pil.<br>Sle.<br>Tach.	Pil.<br>Sle.<br>Tach.	Pil.<br>Sle.<br>Tach.	Pil.<br>Sle.<br>Tach.	Pil.<br>Sle.<br>Tach.	Pil.<br>Sle.<br>Tach.	Pil.<br>Sle.<br>Tach.	+													

		Trem.	Trem.	Trem.	Trem.	Trem.	Trem.	Trem.	Trem.														
	**R9-2000M**	-<br>Sle.<br>Tach.<br>Trem.	-<br>Sle.<br>Tach.<br>Trem.	-	Pil.<br>Sle.<br>Tach.<br>Trem.	Pil.<br>Sle.<br>Tach.<br>Trem.	Pil.<br>Sle.<br>Tach.	Pil.<br>Sle.<br>Tach.	Pil.<br>Sle.<br>Tach.	Pil.	Pil.	-											
	**R10-2000F**	Sle.<br>Tach.<br>Trem.	Pil.<br>Sle.<br>Tach.<br>Trem.	Cat.<br>Dys.<br>Pil.<br>Sle.<br>Tach.<br>Trem.	Cat.<br>Dys.<br>Pil.<br>Sle.<br>Tach.<br>Trem.	Cat.<br>Dys.<br>Pil.<br>Sle.<br>Tach.<br>Trem.	Pil.<br>Sle.<br>Tach.	Pil.<br>Sle.<br>Tach.	Pil.<br>Sle.<br>Tach.	Pil.<br>Sle.<br>Tach.	Pil.	-											
**Caption:**			- Piloerection: Pil.<br>- Sleep: Sle.<br>- Tachypnea: Tach.<br>- Tremors: Trem.<br>- Catalepsy: Cat.<br>- Dyspnea: Dys.<br>- -: Absence of sign or symptom<br>- +: Specimen Death																				

NOTE:

- **R1-2000F:** The presence of tachypnea began immediately upon administration of the substance. Signs such as piloerection, sleepiness and tremors began 30 minutes after administration of the substance.
- **R2-2000F:** The presence of tachypnea began immediately upon administration of the substance. Signs such as piloerection, sleepiness and tremors began 30 minutes after administration of the substance.
- **R3-2000M:** The presence of tachypnea began within 2 minutes of administering the substance. Signs such as piloerection, tremors, began 30 minutes after administration of the substance; while sleep manifested itself 2 hours after the substance to be tested was administered.
- **R4-2000M:** The presence of tachypnea began immediately after administration of the substance. Signs such as piloerection and tremors occurred 30 minutes after the substance was administered, unlike sleep, which began 3 hours after the administration of the substance and ended at 6 p.m.
- **R5-2000F:** The presence of tachypnea, piloerection, tremors, and drowsiness began within 30 minutes of administration of the substance.
- **R6-2000F:** The presence of tachypnea began immediately after administration of the substance. The presence of tremors and sleepiness began 30 minutes after the substance was administered. The specimen showed the presence of piloerection from minute 30, but it was mild during the evaluation time.
- **R7-2000M:** The presence of tachypnea began immediately upon administration of the substance. The presence of tremors, piloerection and sleepiness began 30 minutes after the substance was administered. However, signs such as tremors were not observed 2 hours after the substance was administered, and it was observed that, at 3 hours, the specimen showed this sign again.
- **R8-2000M:** The presence of tachypnea began immediately upon administration of the substance. The presence of tremors, piloerection and sleepiness began 30 minutes after the substance was administered. It was observed that the piloerection was mild 2 hours after the substance was administered, however, it increased progressively during the hours, until 6 p.m..
- **R9-2000M:** The presence of tachypnea began immediately upon administration of the substance. The presence of tremors, piloerection and sleepiness began 30 minutes after the substance was administered. However, 2 hours after the substance was administered, the specimen did not show any of the aforementioned signs, and it was observed that, at 3 hours, the specimen showed the signs again, of which the piloerection was maintained until day 2; sleep and tachypnea until 6 p.m.; and tremors up to 4 hours.
- **R10-2000F:** The presence of piloerection, tachypnea and tremors began 30 minutes after the substance was administered, in the case of piloerection it was observed that the specimen remained with this sign until day 2; in the case of tachypnea, it was observed that the specimen to be evaluated remained with said sign until 24 hours after the administration of the substance; while, in the case of the presence of tremors, they were only observed up to 4 hours after the administration of the vegetable drug. In the case of sleep, it appeared from the time of administration of the plant extract, and was maintained until 24 hours. In the case of catalepsy and dyspnea, they were observed from 2 hours after administration, and maintained until 4 hours.

Reflex Evaluation

Dosage (mg/kg)	Specimen Code	Reflex	Effects After Dosing (Hours)								Effects After Dosage (Days)													
			½	1	2	3	4	10	12	18	1	2	3	4	5	6	7	8	9	10	11	12	13	14
2000 mg/kg	**R1-2000F**	Pain Reflex	N	N	N	A	A	A	A	A	+													
		Corneal Reflex	N	N	N	A	A	A	A	A	+													
		Grip Reflex	N	A	A	A	A	A	A	A	+													
		Straightening Reflex	N	A	A	A	A	A	A	A	+													
		Escape Reflex	A	A	A	A	A	A	A	A	+													
	**R2-2000F**	Pain Reflex	N	N	N	N	N	N	N	N	N	N	N	N	N	N	N	N	N	N	N	N	N	N
		Corneal Reflex	N	N	N	N	N	N	N	N	N	N	N	N	N	N	N	N	N	N	N	N	N	N
		Grip Reflex	N	A	N	N	N	N	N	N	N	N	N	N	N	N	N	N	N	N	N	N	N	N
		Straightening Reflex	N	N	N	N	N	N	N	N	N	N	N	N	N	N	N	N	N	N	N	N	N	N
		Escape Reflex	N	A	N	N	N	N	N	N	N	N	N	N	N	N	N	N	N	N	N	N	N	N
	**R3-2000M**	Pain Reflex	N	A	A	A	A	N	N	N	N	N	N	N	N	N	N	N	N	N	N	N	N	N
		Corneal Reflex	N	N	N	N	N	N	N	N	N	N	N	N	N	N	N	N	N	N	N	N	N	N
		Grip Reflex	N	A	N	N	N	N	N	N	N	N	N	N	N	N	N	N	N	N	N	N	N	N
		Straightening Reflex	N	N	N	N	N	N	N	N	N	N	N	N	N	N	N	N	N	N	N	N	N	N
		Escape Reflex	N	N	N	N	N	N	N	N	N	N	N	N	N	N	N	N	N	N	N	N	N	N
	**R4-2000M**	Pain Reflex	N	A	A	A	A	N	N	N	N	N	N	N	N	N	N	N	N	N	N	N	N	N
		Corneal Reflex	N	N	N	N	N	N	N	N	N	N	N	N	N	N	N	N	N	N	N	N	N	N
		Grip Reflex	N	A	N	N	N	N	N	N	N	N	N	N	N	N	N	N	N	N	N	N	N	N
		Straightening Reflex	N	N	N	N	N	N	N	N	N	N	N	N	N	N	N	N	N	N	N	N	N	N
		Escape Reflex	N	N	N	N	N	N	N	N	N	N	N	N	N	N	N	N	N	N	N	N	N	N
	**R5-2000F**	Pain Reflex	N	A	A	A	A	N	N	N	N	N	N	N	N	N	N	N	N	N	N	N	N	N
		Corneal Reflex	N	N	N	N	N	N	N	N	N	N	N	N	N	N	N	N	N	N	N	N	N	N
		Grip Reflex	N	N	N	N	N	N	N	N	N	N	N	N	N	N	N	N	N	N	N	N	N	N
		Straightening Reflex	N	N	N	N	N	N	N	N	N	N	N	N	N	N	N	N	N	N	N	N	N	N
		Escape Reflex	N	N	N	N	N	N	N	N	N	N	N	N	N	N	N	N	N	N	N	N	N	N
	**R6-2000F**	Pain Reflex	N	A	A	A	A	N	N	N	N	N	N	N	N	N	N	N	N	N	N	N	N	N

		Corneal Reflex	N	N	N	N	N	N	N	N	N	N	N	N	N	N	N	N	N	N	N	N	N	N
		Grip Reflex	N	A	A	A	A	N	N	N	N	N	N	N	N	N	N	N	N	N	N	N	N	N
		Straightening Reflex	N	A	A	A	A	N	N	N	N	N	N	N	N	N	N	N	N	N	N	N	N	N
		Escape Reflex	N	A	A	A	A	N	N	N	N	N	N	N	N	N	N	N	N	N	N	N	N	N
	**R7-2000M**	Pain Reflex	A	A	A	A	A	N	N	N	N	N	N	N	N	N	N	N	N	N	N	N	N	N
		Corneal Reflex	N	N	N	N	N	N	N	N	N	N	N	N	N	N	N	N	N	N	N	N	N	N
		Grip Reflex	A	A	N	N	N	N	N	N	N	N	N	N	N	N	N	N	N	N	N	N	N	N
		Straightening Reflex	A	A	N	N	N	N	N	N	N	N	N	N	N	N	N	N	N	N	N	N	N	N
		Escape Reflex	A	A	A	N	N	N	N	N	N	N	N	N	N	N	N	N	N	N	N	N	N	N
	**R8-2000M**	Pain Reflex	A	A	A	A	A	A	A	A	+													
		Corneal Reflex	N	N	N	A	A	A	A	A	+													
		Grip Reflex	A	A	N	N	N	N	N	N	+													
		Straightening Reflex	A	A	A	A	A	A	A	A	+													
		Escape Reflex	A	A	A	A	A	A	A	A	+													
	**R9-2000M**	Pain Reflex	N	A	A	A	A	N	N	N	N	N	N	N	N	N	N	N	N	N	N	N	N	N
		Corneal Reflex	N	N	N	N	N	N	N	N	N	N	N	N	N	N	N	N	N	N	N	N	N	N
		Grip Reflex	A	N	N	N	N	N	N	N	N	N	N	N	N	N	N	N	N	N	N	N	N	N
		Straightening Reflex	A	N	N	N	N	N	N	N	N	N	N	N	N	N	N	N	N	N	N	N	N	N
		Escape Reflex	A	N	N	N	N	N	N	N	N	N	N	N	N	N	N	N	N	N	N	N	N	N
	**R10-2000F**	Pain Reflex	N	A	A	A	A	N	N	N	N	N	N	N	N	N	N	N	N	N	N	N	N	N
		Corneal Reflex	N	N	N	N	N	N	N	N	N	N	N	N	N	N	N	N	N	N	N	N	N	N
		Grip Reflex	N	A	A	A	A	N	N	N	N	N	N	N	N	N	N	N	N	N	N	N	N	N
		Straightening Reflex	N	A	A	A	A	N	N	N	N	N	N	N	N	N	N	N	N	N	N	N	N	N
		Escape Reflex	N	A	A	A	A	N	N	N	N	N	N	N	N	N	N	N	N	N	N	N	N	N

N: Normal

A: Altered

+: Specimen Death

Evaluation of aspects

Dosage (mg/kg)	Specimen Code	Aspects	Effects After Dosing (Hours)								Effects After Dosage (Days)													
			½	1	2	3	4	10	12	18	1	2	3	4	5	6	7	8	9	10	11	12	13	14
2000 mg/kg	**R1-2000F**	General appearance	A	A	A	A	A	A	A	A	+													
		Tail Appearance	N	N	N	N	N	N	N	N	+													
		Appearance of the eyes	A	A	A	A	A	A	A	A	+													
		Appearance of the genitals	N	N	N	N	N	N	N	N	+													
		Appearance of the stool	N	N	N	N	N	N	N	N	+													
		Urine appearance	N	N	N	N	N	N	N	N	+													
		Gait characteristics	A	A	A	A	A	A	A	A	+													
	**R2-2000F**	General appearance	N	N	N	N	N	N	N	N	N	N	N	N	N	N	N	N	N	N	N	N	N	N
		Tail Appearance	N	N	N	N	N	N	N	N	N	N	N	N	N	N	N	N	N	N	N	N	N	N
		Appearance of the eyes	N	A	A	A	A	A	N	N	N	N	N	N	N	N	N	N	N	N	N	N	N	N
		Appearance of the genitals	N	N	N	N	N	N	N	N	N	N	N	N	N	N	N	N	N	N	N	N	N	N
		Appearance of the stool	N	N	N	N	N	N	N	N	N	N	N	N	N	N	N	N	N	N	N	N	N	N
		Urine appearance	N	N	N	N	N	N	N	N	N	N	N	N	N	N	N	N	N	N	N	N	N	N
		Gait characteristics	N	N	N	N	N	N	N	N	N	N	N	N	N	N	N	N	N	N	N	N	N	N
	**R3-2000M**	General appearance	A	A	A	A	A	A	A	A	A	A	N	N	N	N	N	N	N	N	N	N	N	N
		Tail Appearance	N	N	N	N	N	N	N	N	N	N	N	N	N	N	N	N	N	N	N	N	N	N
		Appearance of the eyes	N	A	A	A	A	A	A	A	A	N	N	N	N	N	N	N	N	N	N	N	N	N
		Appearance of the genitals	N	N	N	N	N	N	N	N	N	N	N	N	N	N	N	N	N	N	N	N	N	N
		Appearance of the stool	N	N	N	N	N	N	N	N	N	N	N	N	N	N	N	N	N	N	N	N	N	N
		Urine appearance	N	N	N	N	N	N	N	N	N	N	N	N	N	N	N	N	N	N	N	N	N	N
		Gait characteristics	N	N	N	N	N	N	N	N	N	N	N	N	N	N	N	N	N	N	N	N	N	N
	**R4-2000M**	General appearance	N	A	A	A	A	A	A	A	A	A	N	N	N	N	N	N	N	N	N	N	N	N
		Tail Appearance	N	N	N	N	N	N	N	N	N	N	N	N	N	N	N	N	N	N	N	N	N	N
		Appearance of the eyes	N	A	A	A	A	A	A	A	A	N	N	N	N	N	N	N	N	N	N	N	N	N
		Appearance of the genitals	N	N	N	N	N	N	N	N	N	N	N	N	N	N	N	N	N	N	N	N	N	N
		Appearance of the stool	N	N	N	N	N	N	N	N	N	N	N	N	N	N	N	N	N	N	N	N	N	N

		Urine appearance	N	N	N	N	N	N	N	N	N	N	N	N	N	N	N	N	N	N	N	N	N	N
		Gait characteristics	N	N	N	N	N	N	N	N	N	N	N	N	N	N	N	N	N	N	N	N	N	N
	**R5-2000F**	General appearance	N	N	A	A	A	A	A	A	A	A	N	N	N	N	N	N	N	N	N	N	N	N
		Tail Appearance	N	N	N	N	N	N	N	N	N	N	N	N	N	N	N	N	N	N	N	N	N	N
		Appearance of the eyes	N	A	A	A	A	A	A	A	A	N	N	N	N	N	N	N	N	N	N	N	N	N
		Appearance of the genitals	N	N	N	N	N	N	N	N	N	N	N	N	N	N	N	N	N	N	N	N	N	N
		Appearance of the stool	N	N	N	N	N	N	N	N	N	N	N	N	N	N	N	N	N	N	N	N	N	N
		Urine appearance	N	N	N	N	N	N	N	N	N	N	N	N	N	N	N	N	N	N	N	N	N	N
		Gait characteristics	N	N	N	N	N	N	N	N	N	N	N	N	N	N	N	N	N	N	N	N	N	N
	**R6-2000F**	General appearance	A	A	A	A	A	A	A	A	A	A	N	N	N	N	N	N	N	N	N	N	N	N
		Tail Appearance	N	N	N	N	N	N	N	N	N	N	N	N	N	N	N	N	N	N	N	N	N	N
		Appearance of the eyes	A	A	A	A	A	A	A	A	A	N	N	N	N	N	N	N	N	N	N	N	N	N
		Appearance of the genitals	N	N	N	N	N	N	N	N	N	N	N	N	N	N	N	N	N	N	N	N	N	N
		Appearance of the stool	N	N	N	N	N	N	N	N	N	N	N	N	N	N	N	N	N	N	N	N	N	N
		Urine appearance	N	N	N	N	N	N	N	N	N	N	N	N	N	N	N	N	N	N	N	N	N	N
		Gait characteristics	N	N	N	A	A	A	A	A	A	N	N	N	N	N	N	N	N	N	N	N	N	N
	**R7-2000M**	General appearance	A	A	A	A	A	A	A	A	A	A	N	N	N	N	N	N	N	N	N	N	N	N
		Tail Appearance	N	N	N	N	N	N	N	N	N	N	N	N	N	N	N	N	N	N	N	N	N	N
		Appearance of the eyes	A	A	A	A	A	A	A	A	A	N	N	N	N	N	N	N	N	N	N	N	N	N
		Appearance of the genitals	N	N	N	N	N	N	N	N	N	N	N	N	N	N	N	N	N	N	N	N	N	N
		Appearance of the stool	N	N	N	N	N	N	N	N	N	N	N	N	N	N	N	N	N	N	N	N	N	N
		Urine appearance	N	N	N	N	N	N	N	N	N	N	N	N	N	N	N	N	N	N	N	N	N	N
		Gait characteristics	N	N	N	N	N	A	A	A	A	N	N	N	N	N	N	N	N	N	N	N	N	N
	**R8-2000M**	General appearance	A	A	A	A	A	A	A	A	+													
		Tail Appearance	N	N	N	N	N	N	N	N	+													
		Appearance of the eyes	A	A	A	A	A	A	A	A	+													
		Appearance of the genitals	N	N	N	N	N	N	N	N	+													
		Appearance of the stool	N	N	N	N	N	N	N	N	+													
		Urine appearance	N	N	N	N	N	N	N	N	+													
		Gait characteristics	N	N	N	N	N	N	N	N	+													

	**R9-2000M**	General appearance	A	N	N	A	A	A	A	A	A	A	N	N	N	N	N	N	N	N	N	N	N	N
		Tail Appearance	N	N	A	N	N	N	N	N	N	N	N	N	N	N	N	N	N	N	N	N	N	N
		Appearance of the eyes	A	N	N	A	A	A	A	A	A	N	N	N	N	N	N	N	N	N	N	N	N	N
		Appearance of the genitals	N	N	N	N	N	N	N	N	N	N	N	N	N	N	N	N	N	N	N	N	N	N
		Appearance of the stool	N	N	N	N	N	N	N	N	N	N	N	N	N	N	N	N	N	N	N	N	N	N
		Urine appearance	N	N	N	N	N	N	N	N	N	N	N	N	N	N	N	N	N	N	N	N	N	N
		Gait characteristics	N	N	N	N	N	N	N	N	N	N	N	N	N	N	N	N	N	N	N	N	N	N
	**R10-2000F**	General appearance	A	A	A	A	A	A	A	A	A	A	N	N	N	N	N	N	N	N	N	N	N	N
		Tail Appearance	N	N	N	N	N	N	N	N	N	N	N	N	N	N	N	N	N	N	N	N	N	N
		Appearance of the eyes	A	A	A	A	A	A	A	A	A	N	N	N	N	N	N	N	N	N	N	N	N	N
		Appearance of the genitals	N	N	N	N	N	N	N	N	N	N	N	N	N	N	N	N	N	N	N	N	N	N
		Appearance of the stool	N	N	N	N	N	N	N	N	N	N	N	N	N	N	N	N	N	N	N	N	N	N
		Urine appearance	N	N	N	N	N	N	N	N	N	N	N	N	N	N	N	N	N	N	N	N	N	N
		Gait characteristics	N	N	N	A	A	A	A	A	A	N	N	N	N	N	N	N	N	N	N	N	N	N

N: Normal

A: Altered

+: Specimen Death

NOTE:

- **R1-2000F:** Mouse coat and position characteristics related to acute pain. Presence of slanted eyes. Regarding the characteristics of the gait, it was slow and with little motor coordination.
- **R2-2000F:** Presence of slanted eyes.
- **R3-2000M:** The specimen had a triangular snout and slightly slanted eyes.
- **R4-2000M** The specimen featured a triangular snout and slanted eyes.
- **R5-2000F:** The specimen had a triangular snout and slanted eyes.
- **R6-2000F:** Characteristics of fur and mouse position referring to mild pain. Presence of slanted eyes. Regarding the characteristics of the gait, it was slow and with little motor coordination.
- **R7-2000M:** Coat and mouse position characteristics referring to mild pain. Presence of slanted eyes. Regarding the characteristics of the gait, it was slow and with little motor coordination.
- **R8-2000M:** Characteristics of fur and mouse position referring to acute pain. Presence of slanted eyes. Regarding the characteristics of the gait, it was normal.
- **R9-2000M:** The specimen had a triangular snout and slightly slanted eyes, during the 30 minutes after the substance was administered, however, these aspects normalized until 2 hours; while after 3 hours, these aspects were progressively increased until day 2 and 1, respectively. The presence of an "S" shaped glue was observed 2 hours after the substance was administered.
- **R10-2000F:** Coat characteristics and position of the specimen referring to severe pain, with presence of slanted eyes. As for the characteristics of the gait, it was slow and with little motor coordination.

Organ weights at necropsy

Organ	Specimen										Promedio
	R1-2000F	R2-2000F	R3-2000M	R4-2000M	R5-2000F	R6-2000F	R7-2000M	R8-2000M	R9-2000M	R10-2000F	
**Liver**	2.7473 g	1.9623 g	1.7980 g	2.8122 g	1.7171 g	2.5366 g	2.1972 g	1.8719 g	2.1423 g	1.9925 g	2.0954 g
**Kidneys**	0.6086 g	0.5830 g	0.6158 g	0.7557 g	0.6003 g	0.7426 g	0.8408 g	0.5694 g	0.6087 g	0.4970 g	0.5776 g
**Spleen**	0.1817 g	0.2742 g	0.1268 g	0.1663 g	0.1599 g	0.2667 g	0.1509 g	0.1174 g	0.1826 g	0.6031 g	0.3919 g
**Heart**	0.1933 g	0.1623 g	0.2216 g	0.2435 g	0.1585 g	0.2655 g	0.2581 g	0.2119 g	0.2008 g	0.1593 g	0.1861 g
**Lung**	0.6347 g	0.3864 g	0.3757 g	0.4681 g	0.4317 g	0.5038 g	0.5613 g	0.5293 g	0.4619 g	0.3709 g	0.4273 g
**Stomach**	0.4521 g	0.5868 g	0.6946 g	0.9736 g	0.5930 g	0.7213 g	0.6296 g	0.5035 g	0.5096 g	0.4521 g	0.5407 g
**Brain**	0.3263 g	0.3928 g	0.3958 g	0.3759 g	0.4338 g	0.4174 g	0.4182 g	0.3397 g	0.4151 g	0.4821 g	0.4363 g
**Female gonads**	0.2405 g	0.2798 g	-	-	0.1340 g	0.4136 g	-	-	-	0.4539 g	0.3604 g
**Male gonads**	-	-	1.0348 g	1.0886 g	-	-	1.1783 g	0.8144 g	0.8479 g	-	0.9928 g

Macroscopic observations of organs at necropsy

Organ	Specimen									
	**R1-2000F**	**R2-2000F**	**R3-2000M**	**R4-2000M**	**R5-2000F**	**R6-2000F**	**R7-2000M**	**R8-2000M**	**R9-2000M**	**R10-2000F**
**Liver**	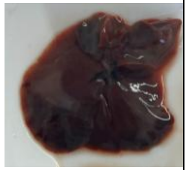 No abnormalities detected	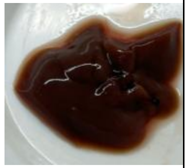 No abnormalities detected	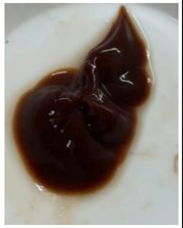 No abnormalities detected	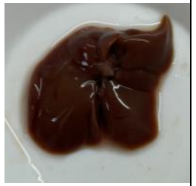 No abnormalities detected	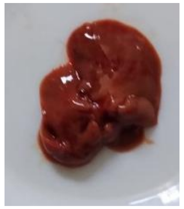 No abnormalities detected	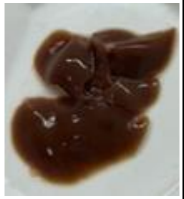 No abnormalities detected	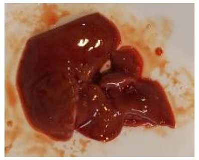 No abnormalities detected	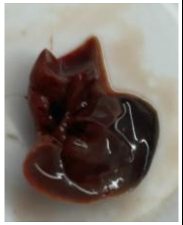 No abnormalities detected	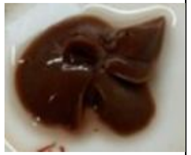 No abnormalities detected	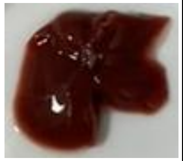 No abnormalities detected
**Kidneys**	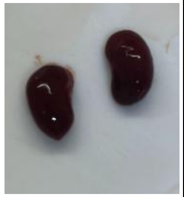 No abnormalities detected	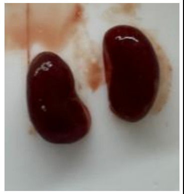 No abnormalities detected	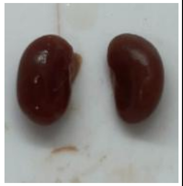 No abnormalities detected	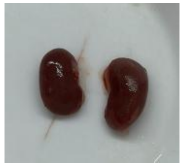 No abnormalities detected	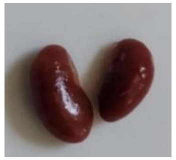 No abnormalities detected	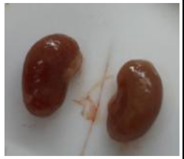 No abnormalities detected	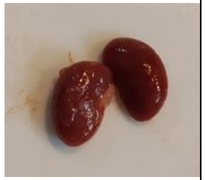 No abnormalities detected	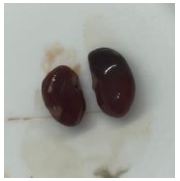 No abnormalities detected	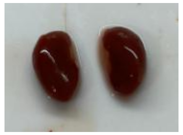 No abnormalities detected	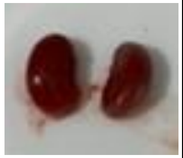 No abnormalities detected
**Spleen**										

	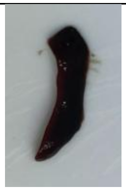 No abnormalities detected	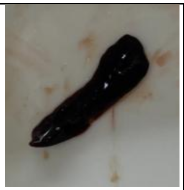 No abnormalities detected	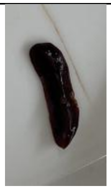 No abnormalities detected	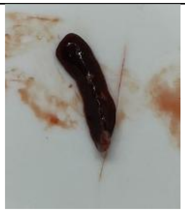 No abnormalities detected	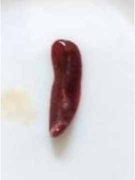 No abnormalities detected	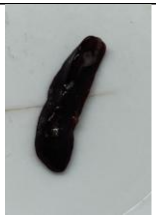 No abnormalities detected	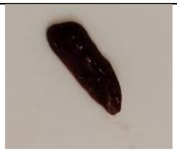 No abnormalities detected	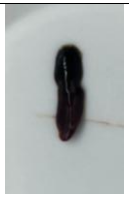 No abnormalities detected	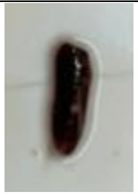 No abnormalities detected	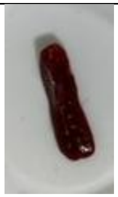 Mega Spleen
**Heart**	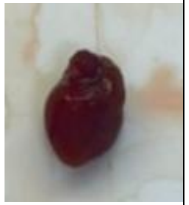 No abnormalities detected	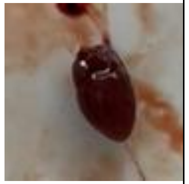 No abnormalities detected	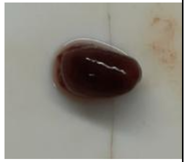 No abnormalities detected	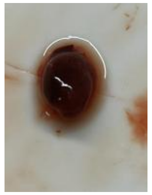 No abnormalities detected	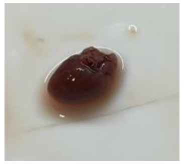 No abnormalities detected	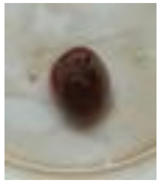 No abnormalities detected	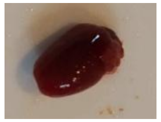 No abnormalities detected	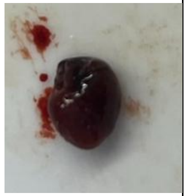 No abnormalities detected	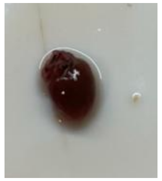 No abnormalities detected	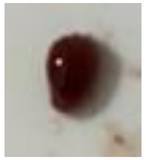 No abnormalities detected
**Lung**	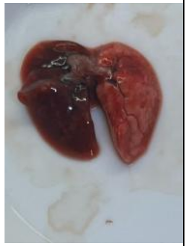 Right lung with greater presence of oxygen.	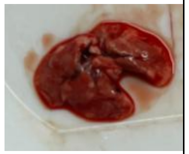 No abnormalities detected	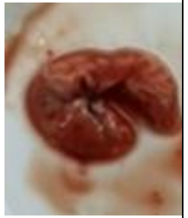 No abnormalities detected	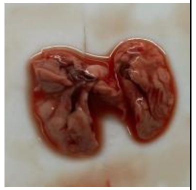 No abnormalities detected	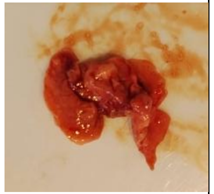 No abnormalities detected	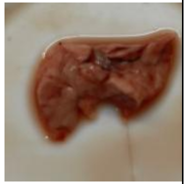 No abnormalities detected	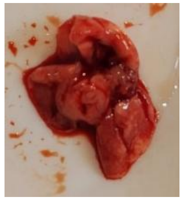 No abnormalities detected	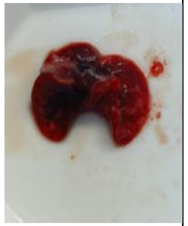 Right lung with greater presence of oxygen.	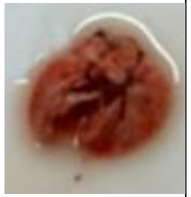 No abnormalities detected	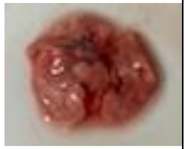 No abnormalities detected

**Stomach**	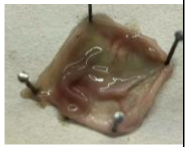<br>The characteristic roughness of the organ was not observed.	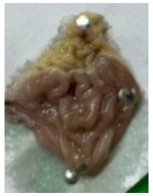<br>No abnormalities detected	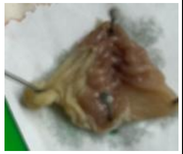<br>No abnormalities detected	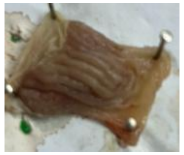<br>No abnormalities detected	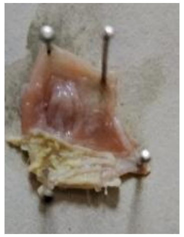<br>No abnormalities detected	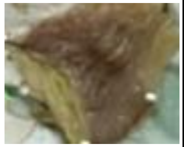<br>No abnormalities detected	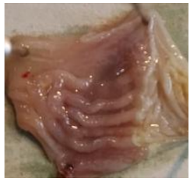<br>No abnormalities detected	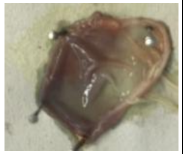<br>No abnormalities detected	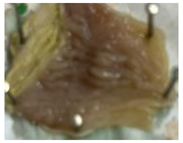<br>No abnormalities detected	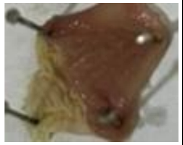<br>No abnormalities detected
**Brain**	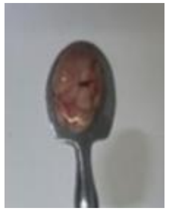<br>No abnormalities detected	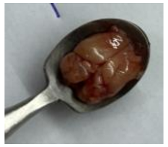<br>No abnormalities detected	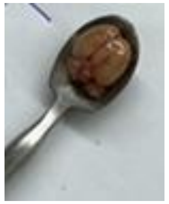<br>No abnormalities detected	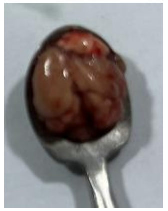<br>No abnormalities detected	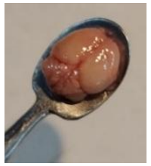<br>No abnormalities detected	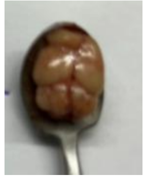<br>No abnormalities detected	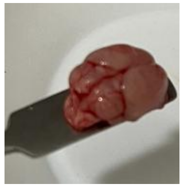<br>No abnormalities detected	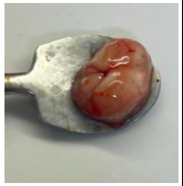<br>No abnormalities detected	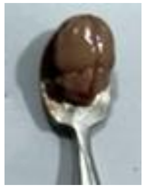<br>No abnormalities detected	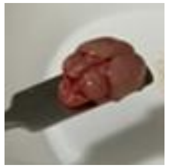<br>No abnormalities detected

**Gonads**	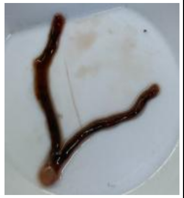 No abnormalities detected	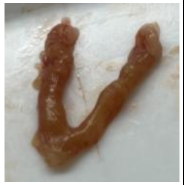 No abnormalities detected	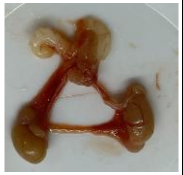 No abnormalities detected	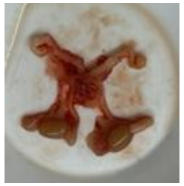 No abnormalities detected	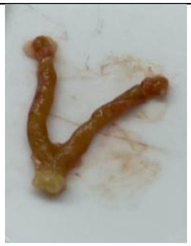 No abnormalities detected	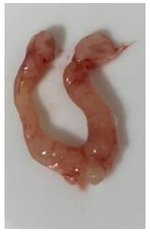 No abnormalities detected	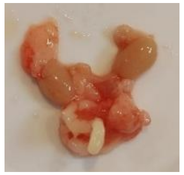 No abnormalities detected	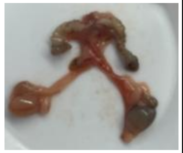 No abnormalities detected	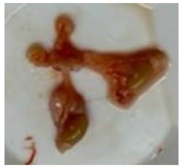 No abnormalities detected	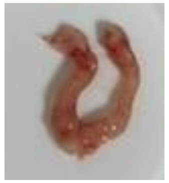 No abnormalities detected
**Intestin e**	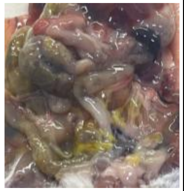 Presence of viscous yellow matter.	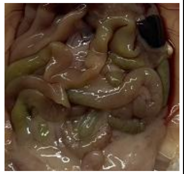 No abnormalities detected	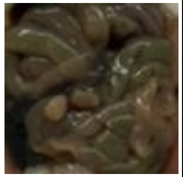 No abnormalities detected	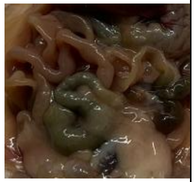 No abnormalities detected	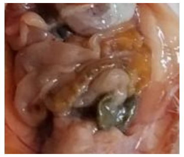 No abnormalities detected	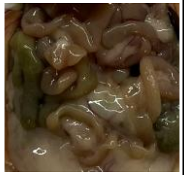 No abnormalities detected	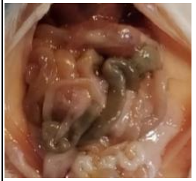 No abnormalities detected	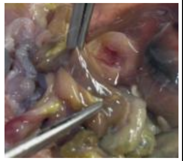 Presencia de materia amarilla viscosa.	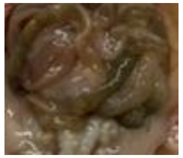 No abnormalities detected	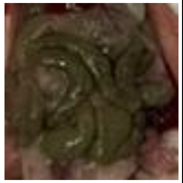 No abnormalities detected
